# Metagenomic insights into the effects of submerged plants on functional potential of microbial communities in wetland sediments

**DOI:** 10.1007/s42995-021-00100-3

**Published:** 2021-08-27

**Authors:** Binhao Wang, Xiafei Zheng, Hangjun Zhang, Xiaoli Yu, Yingli Lian, Xueqin Yang, Huang Yu, Ruiwen Hu, Zhili He, Fanshu Xiao, Qingyun Yan

**Affiliations:** 1 Environmental Microbiomics Research Center, School of Environmental Science and Engineering, Southern Marine Science and Engineering Guangdong Laboratory (Zhuhai), Sun Yat-Sen University, Guangzhou, 510006 China; 2 Institute of Soil and Water Resources and Environmental Science, College of Environmental and Resource Sciences, Zhejiang University, Hangzhou, 310058 China; 3 College of Life and Environmental Sciences, Hangzhou Normal University, Hangzhou, 310036 China; 4 College of Agronomy, Hunan Agricultural University, Changsha, 410128 China

**Keywords:** Submerged plants, Metagenome, Elemental cycling, Methanogenesis, *Verstraetearchaeota*

## Abstract

Submerged plants in wetlands play important roles as ecosystem engineers to improve self-purification and promote elemental cycling. However, their effects on the functional capacity of microbial communities in wetland sediments remain poorly understood. Here, we provide detailed metagenomic insights into the biogeochemical potential of microbial communities in wetland sediments with and without submerged plants (i.e., *Vallisneria natans*). A large number of functional genes involved in carbon (C), nitrogen (N) and sulfur (S) cycling were detected in the wetland sediments. However, most functional genes showed higher abundance in sediments with submerged plants than in those without plants. Based on the comparison of annotated functional genes in the N and S cycling databases (i.e., NCycDB and SCycDB), we found that genes involved in nitrogen fixation (e.g., *nifD/H/K/W*), assimilatory nitrate reduction (e.g., *nasA* and *nirA*), denitrification (e.g., *nirK/S* and *nosZ*), assimilatory sulfate reduction (e.g., *cysD/H/J/N/Q* and *sir*), and sulfur oxidation (e.g., *glpE, soeA, sqr* and *sseA*) were significantly higher (corrected *p* < 0.05) in vegetated vs. unvegetated sediments. This could be mainly driven by environmental factors including total phosphorus, total nitrogen, and C:N ratio. The binning of metagenomes further revealed that some archaeal taxa could have the potential of methane metabolism including hydrogenotrophic, acetoclastic, and methylotrophic methanogenesis, which are crucial to the wetland methane budget and carbon cycling. This study opens a new avenue for linking submerged plants with microbial functions, and has further implications for understanding global carbon, nitrogen and sulfur cycling in wetland ecosystems.

**Supplementary Information:**

The online version contains supplementary material available at 10.1007/s42995-021-00100-3.

## Introduction

Wetlands play important roles in global carbon (C), nitrogen (N) and sulfur (S) cycling, and contribute significantly to the processes of methane production (Borrel et al. 2011; Emilson et al. 2018), nitrogen transformation and removal (Harrison et al. 2009; Liu et al. 2018), and sulfur reduction and oxidation (Holmer and Storkholm 2001; Purcell et al. 2014). The resultant ‘hot spots’ of biogeochemical cycling involving plants and microorganisms may create an eco-buffer zone for environmental protection, regulation and management (Chen et al. 2014; Sims et al. 2013). As plant-microbe interactions could considerably influence element cycling in wetland sediments (Kofoed et al. 2012; Vilacosta et al. 2016), studying the effects of plants on the functional potential of microbial communities is very important to understand wetland ecosystem function.

Aquatic macrophytes (e.g., *Vallisneria natans, Hydrilla verticillata* and *Typha latifolia*) are commonly used as biological tools for reducing nitrogen and sulfur elements, and raising dissolved oxygen (DO) concentration in natural or constructed wetlands (Chen et al. 2014; Qiu et al. 2001; Zhu et al. 2016). They almost invariably show positive effects on improving water clarity and increasing the stability of aquatic ecosystems by a variety of different mechanisms (Scheffer et al. 1993; Zhu et al. 2016). Specifically, leaf and root exudates supply nutrients and create micro-niches for microorganisms (Michael et al. 2008; Wiessner et al. 2010) which stimulate ecosystem element cycling (Kaokniffin et al. 2010; Ullah et al. 2014). For example, the oxidation of sedimental NH_4_^+^-N to NO_3_^−^-N could be enhanced by submerged macrophyte rhizosphere microorganisms (Ottosen et al. 1999), which in turn benefit the plants by enhancing the supply of available N (Bodelier et al. 2006). Also, macrophytes could affect sedimental biogeochemical cycling by increasing the activity of microbes, such as methanogens, which would lead to high methane production (Emilson et al. 2018). In turn, the sediment microbial communities play key roles in the health of plants, for example, facilitating the oxidation of phytotoxic sulfide into non-toxic sulfate or protecting plants from pathogens (Frigaard and Dahl 2008; Ugarelli et al. 2019). Therefore, plants may regulate rhizosphere microorganisms to improve their own fitness (Zhalnina et al. 2018).

Although the effects of macrophytes on microbial community structure have been sufficient reports (Liu et al. 2018), further studies are needed to provide more direct insights into how vegetation impacts microbial processes and functions (Grossart et al. 2020). For example, vegetated sediments show a higher abundance of *nirS* and *nirK* genes than unvegetated sediments in oligotrophic shallow lakes, and some nitrogen cycling processes are promoted by vegetation (Vilacosta et al. 2016). Additionally, wetlands are considered to be a significant source of greenhouse gases (e.g., CH_4_ and N_2_O), which also could be regulated by aquatic macrophytes (Heilman and Carlton 2001; Veraart et al. 2011). In contrast, other studies found that submerged macrophytes had little effect on N_2_O production (Liu et al. 2018; Yao et al. 2018). However, the effects of submerged plants on microbial functions in C, N and S cycling have not yet been fully explored. Therefore, it is important to identify molecular markers (e.g., key functional genes involved in biogeochemical cycling) in microorganisms to predict the effects of submerged plants on the functional status and stability of wetland ecosystems.

The submerged macrophyte *Vallisneria natans* is a widely distributed perennial species with well-developed roots that provides valuable ecosystem services to improve water quality and filter nutrients (Qiu et al. 2001). A recent study indicated that *V. natans* can even resist the adverse impacts of algal blooms (Jiang et al. 2019). Although the feedback of microbial community composition to *V. natans* has previously been studied (Wang et al. 2017), it remains elusive how *V. natans* affects the functional potential of microbial groups involved in elemental cycling in wetland sediments. The main aim of this study was to determine the influence of *V. natans* on functional diversity of microbial communities. We hypothesized that submerged plants provide an important impetus (direct or indirect) for accelerating biogeochemical cycling through plant-microbe interactions in wetland sediments. To test this hypothesis, metagenomic analysis was performed to study microbial functions acclimating to *V. natans* in the Xixi National Wetland Park*.* We also reconstructed and characterized draft genomes to identify the main and potential microbial taxa involved in sedimental element cycling. This study thus provides insights to link submerged macrophytes with the functional capacity of sedimental microbial communities in urban wetlands.

## Results

### Metabolic genes involved in nitrogen cycling

**Fig. 1:**
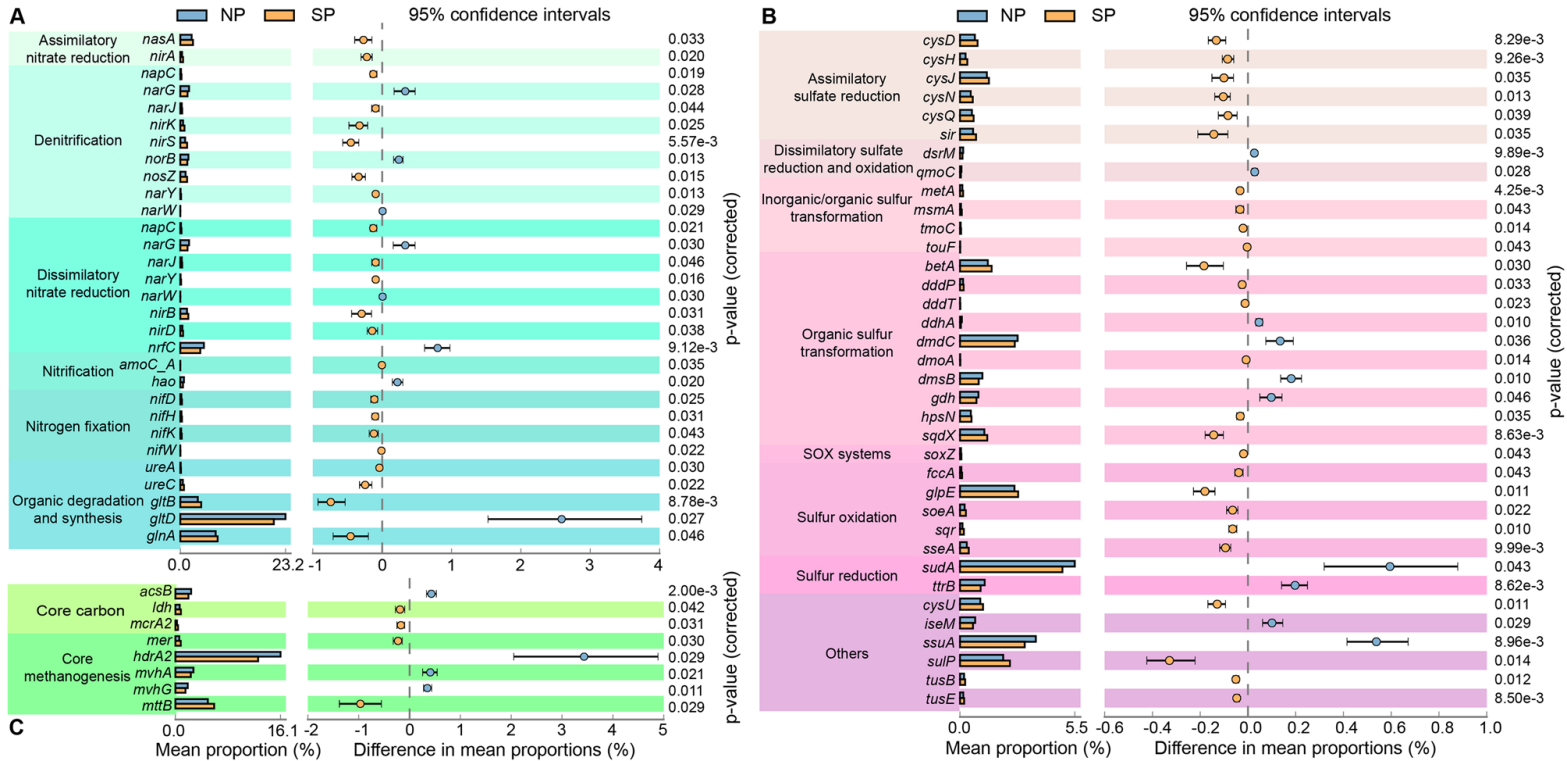
Abundance comparison of key genes involved in nitrogen cycling (**a**), sulfur cycling (**b**), and methanogenesis (**c**) between sediments with (SP) and without (NP) submerged plants. The *p* values are based on Welch’s exact test and corrected by Benjamini-Hochberg false discovery rate (FDR) method

Key genes for microbial nitrogen metabolism were searched for in the metagenomes of two types of sedimental samples (i.e., with submerged plants, SP; with no plants, NP), and differences between SP and NP were clear regarding gene inventories. In total, more than 70 functional genes involved in nitrification, denitrification, assimilatory nitrate reduction, dissimilatory nitrate reduction, nitrogen fixation, anammox, organic degradation and synthesis were annotated in this study (Supplementary file 1). More than two-thirds of the functional genes involved in the pathways of nitrogen fixation, assimilatory nitrate reduction and denitrification were more abundant in the SP sediments than in the NP sediments (Fig. 1a). Specifically, genes encoding the glutamate synthase (NADPH/NADH) small chain (*gltD*) accounted for more than 20% of the total abundance of functional genes involved in the nitrogen cycle and had significantly higher abundance in SP than in NP (corrected *p* < 0.05). Similarly, the abundance of genes encoding enzymes of the nitrate reduction process, including assimilatory nitrate reductase catalytic subunit (*nasA*), ferredoxin-nitrite reductase (*nirA*), and nitrite reductase (NADH) (*nirB/D*), was significantly higher in SP than NP. In contrast, the abundance of genes encoding nitric oxide reductase (*norB*) and hydroxylamine dehydrogenase (*hao*) had significantly higher abundances (corrected *p* < 0.05) in NP than SP. For the nitrogen fixation pathway, genes encoding the nitrogenase molybdenum-iron protein (*nifD*/*K*), nitrogenase iron protein (*nifH*), and nitrogenase-stabilizing/protective protein (*nifW*) were more abundant in SP than NP. In addition, the abundance of genes encoding urease subunit alpha/gamma (*ureC/A*), the key enzyme in the conversion of organic nitrogen to ammonium nitrogen, was higher in the SP than in the NP sediments (Fig. S1 in Supplementary file 2). The correlation variations based on redundancy analysis (RDA) indicated that total phosphorus (TP), total nitrogen (TN) and C:N ratio (C/N) were the major factors shaping microbial communities (Fig. S2a in Supplementary file 2), and explain 59.66% of the community variances by the first two axes. The relationships between environmental factors and microbial functional genes involved in nitrogen metabolic pathways were further investigated by partial Mantel tests. We found the genes involved in assimilatory nitrate reduction (*r* = 0.512), denitrification (*r* = 0.425) and dissimilatory nitrate reduction (*r* = 0.372) were significantly (*p* < 0.05) correlated with C/N (Table 1).

**Table 1:** Summary statistics for partial Mantel tests of correlation between functional genes and environmental factors

Gene category	pH	Conductivity (μs/cm)	TP (g/kg)	TN (g/kg)	TC (g/kg)	C/N
Nitrogen-cycling pathway						
Anammox	− 0.124 (0.792)	− 0.311 (0.954)	0.104 (0.263)	− 0.035 (0.578)	− 0.320 (0.956)	0.110 (0.313)
Assimilatory nitrate reduction	0.254 (0.055)	0.347 (**0.017**)	− 0.220 (0.919)	− 0.438 (0.990)	0.050 (0.412)	0.512 (**0.006**)
Denitrification	0.184 (0.094)	0.380 (**0.014**)	0.009 (0.428)	− 0.027 (0.560)	0.299 (**0.019**)	0.425 (**0.008**)
Dissimilatory nitrate reduction	0.421 (**0.020**)	0.446 (**0.007**)	− 0.198 (0.932)	− 0.086 (0.723)	0.322 (**0.016**)	0.372 (**0.019**)
Nitrification	0.040 (0.384)	0.206 (0.109)	0.266 (0.100)	− 0.076 (0.642)	0.284 (0.077)	0.085 (0.274)
Nitrogen fixation	0.453 (**0.007**)	0.476 (**0.003**)	− 0.270 (0.968)	0.207 (0.151)	0.321 (**0.024**)	0.049 (0.304)
Organic degradation and synthesis	− 0.081 (0.681)	0.093 (0.294)	0.127 (0.248)	− 0.256 (0.915)	− 0.004 (0.505)	0.331 (**0.049**)
Sulfur-cycling pathway						
Assimilatory sulfate reduction	0.122 (0.222)	0.331 (**0.028**)	− 0.208 (0.922)	− 0.361 (0.987)	0.007 (0.511)	0.603 (**0.005**)
Dissimilatory sulfate reduction and oxidation	0.180 (**0.120**)	0.257 (0.069)	− 0.191 (0.838)	− 0.273 (0.961)	− 0.050 (0.603)	0.346 (**0.013**)
Link between inorganic and organic sulfur transformation	− 0.142 (0.812)	0.066 (0.389)	− 0.056 (0.536)	− 0.285 (0.906)	− 0.104 (0.703)	0.426 (0.050)
Organic sulfur transformation	0.026 (0.475)	0.147 (0.238)	− 0.212 (0.878)	− 0.301 (0.946)	− 0.091 (0.703)	0.463 (**0.021**)
SOX systems	− 0.090 (0.717)	0.059 (0.413)	0.038 (0.364)	− 0.349 (0.968)	− 0.012 (0.535)	0.456 (**0.016**)
Sulfur disproportionation	− 0.016 (0.559)	0.069 (0.342)	0.088 (0.268)	− 0.074 (0.676)	0.064 (0.331)	− 0.061 (0.594)
Sulfur oxidation	0.132 (0.167)	0.286 (**0.024**)	− 0.110 (0.751)	− − 0.060 (0.650)	0.097 (0.306)	0.379 (**0.026**)
Sulfur reduction	0.077 (0.354)	0.137 (0.227)	0.122 (0.209)	-0.226 (0.849)	0.128 (0.292)	0.222 (0.139)
Methanogenesis						
Core methanogenesis	− 0.295 (0.977)	− 0.084 (0.692)	0.164 (0.214)	− 0.221 (0.871)	− 0.122 (0.71)	0.313 (0.101)

Values are correlation coefficients with *p* values in brackets (*p* < 0.05 in bold)

TP: Total phosphorus; TC: Total carbon; TN: Total nitrogen; C/N: Total carbon/Total nitrogen

### Metabolic genes involved in sulfur cycling

More than 200 functional genes involved in assimilatory sulfate reduction, sulfate reduction, dissimilatory sulfate reduction and oxidation, sulfur reduction, sulfur oxidization (SOX) systems, sulfur oxidation, sulfur disproportionation, organic sulfur transformation, and the link between inorganic and organic sulfur transformation, were identified in both NP and SP samples (Supplementary file 3). Among them, 26 genes encoding the key enzyme of sulfur cycling showed significantly higher (corrected *p* < 0.05) abundances in the SP than in the NP sediment samples (Fig. 1b). For the assimilatory sulfate reduction pathway, most relevant genes involved in encoding for sulfate adenylyltransferase subunit (*cysD*/*N*), phosphoadenosine phosphosulfate reductase (*cysH*), sulfite reductase (NADPH) flavoprotein alpha-component (*cysJ*) and sulfite reductase (ferredoxin) (*sir*) were significantly more abundant in SP than NP sediments. Also, SP had significantly higher abundances of genes encoding thiosulfate sulfurtransferase (*glpE*), sulfite dehydrogenase (quinone) subunit (*soeA*), sulfide-quinone oxidoreductase (*sqr*), and the key enzyme of sulfide oxidation (Fig. 1b). For the SOX systems, similar gene abundances were found in NP and SP, except for *soxZ*. In contrast, the NP microbial communities showed significantly higher (corrected *p* < 0.05) abundances of genes encoding 3-(methylthio)propanoyl-CoA dehydrogenase (dmdC; catalyzing organic sulfur oxidation), anaerobic dimethyl sulfoxide reductase subunit B (*dmsB*) and sulfide dehydrogenase subunit alpha (*sudA*). According to the RDA plots, the environmental factors TN, TP and C/N had significant influences on the relative abundances of functional genes involved in sulfur cycling, and explained 58.07% of the total variation by the first two axes (Fig. S2b in Supplementary file 2). Furthermore, C/N showed significant positive correlations with the functional genes involved in assimilatory sulfate reduction (*r* = 0.603, *p* < 0.05), dissimilatory sulfate reduction and oxidation (*r* = 0.346, *p* < 0.05), organic sulfur transformation (*r* = 0.463, *p* < 0.05), SOX systems (*r* = 0.456, *p* < 0.05), and the sulfur oxidation pathway (*r* = 0.379, *p* < 0.05) (Table 1).

### Metabolic genes and potential pathways involved in methanogenesis

Genes involved in methanogenesis are summarized in Supplementary file 4. The detected methanogenic processes included methanol, methylamine, acetate, carbon dioxide, core carbon and core methanogenesis. Gene encoding for the methyl coenzyme M reductase system, component A2 (*mcrA2*/K00400) was more abundant in the SP than NP sediments. Similarly, abundances of genes encoding the trimethylamine-corrinoid protein Co-methyltransferase (*mttB*) and 5,10-methylenetetra-hydro-methanopterin reductase (*mer*) were significantly higher (corrected *p* < 0.05) in SP sediments than in NP sediments. In contrast, the abundance of gene associated with heterodisulfide reductase subunit A2 (*hdrA2*) was significantly higher in the NP than the SP sediments (corrected *p* < 0.05) (Fig. 1c).

**Fig. 2:**
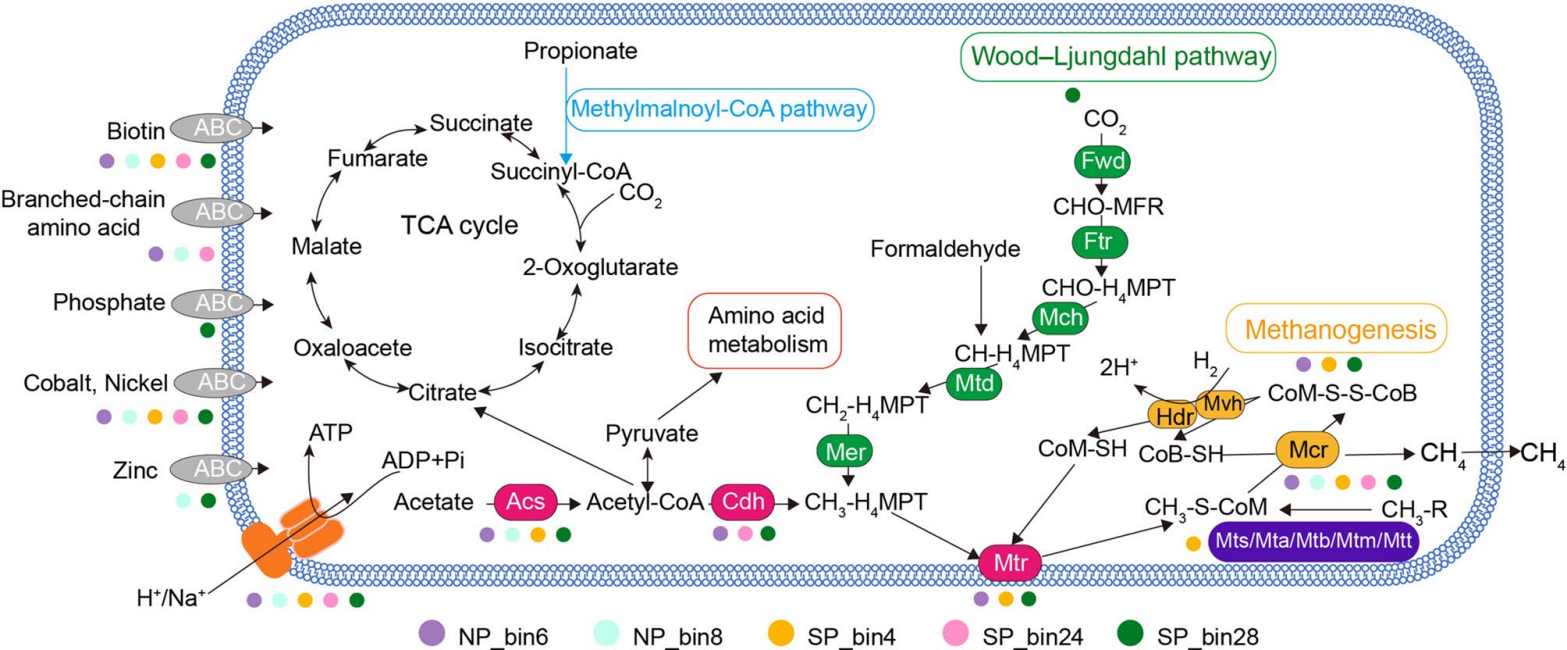
Predicted methanogenic pathways in the archaeal lineages based on the gene analysis of five MAGs with completeness > 80%. Genes involved in methanogenesis and various transporters are shown in different colors

To reveal the diversity of metabolic potential for methanogenesis, we chose relatively high-quality genome bins (completeness > 80%) and performed bin annotation to identify the metabolic pathways of hydrogenotrophic, acetoclastic, and methylotrophic methanogenesis in urban wetland sediments (Fig. 2). Specifically, SP_bin28 had genes encoding conserved core enzymes of hydrogenotrophic methanogenesis, including Fwd, Ftr, Mch, Mtd, Mer, Mtr and Mcr. NP_bin6 and SP_bin28 contained genes encoding Acs, Cdh, Mtr, and Mcr enzymes for the utilization of acetate, which showed potential for acetoclastic methanogenesis. SP_bin4 contained genes encoding methyl-compound methyltransferase, such as Mts, Mta, Mtb, Mtm and Mtt. These genes showed a potential for methane production through the methylotrophic pathway. However, no complete methanogenesis pathways were identified in NP_bin8 and SP_bin24 due to the incompleteness of their genomes.

### Phylogeny of metagenome-assembled genomes (MAGs)

**Fig. 3:**
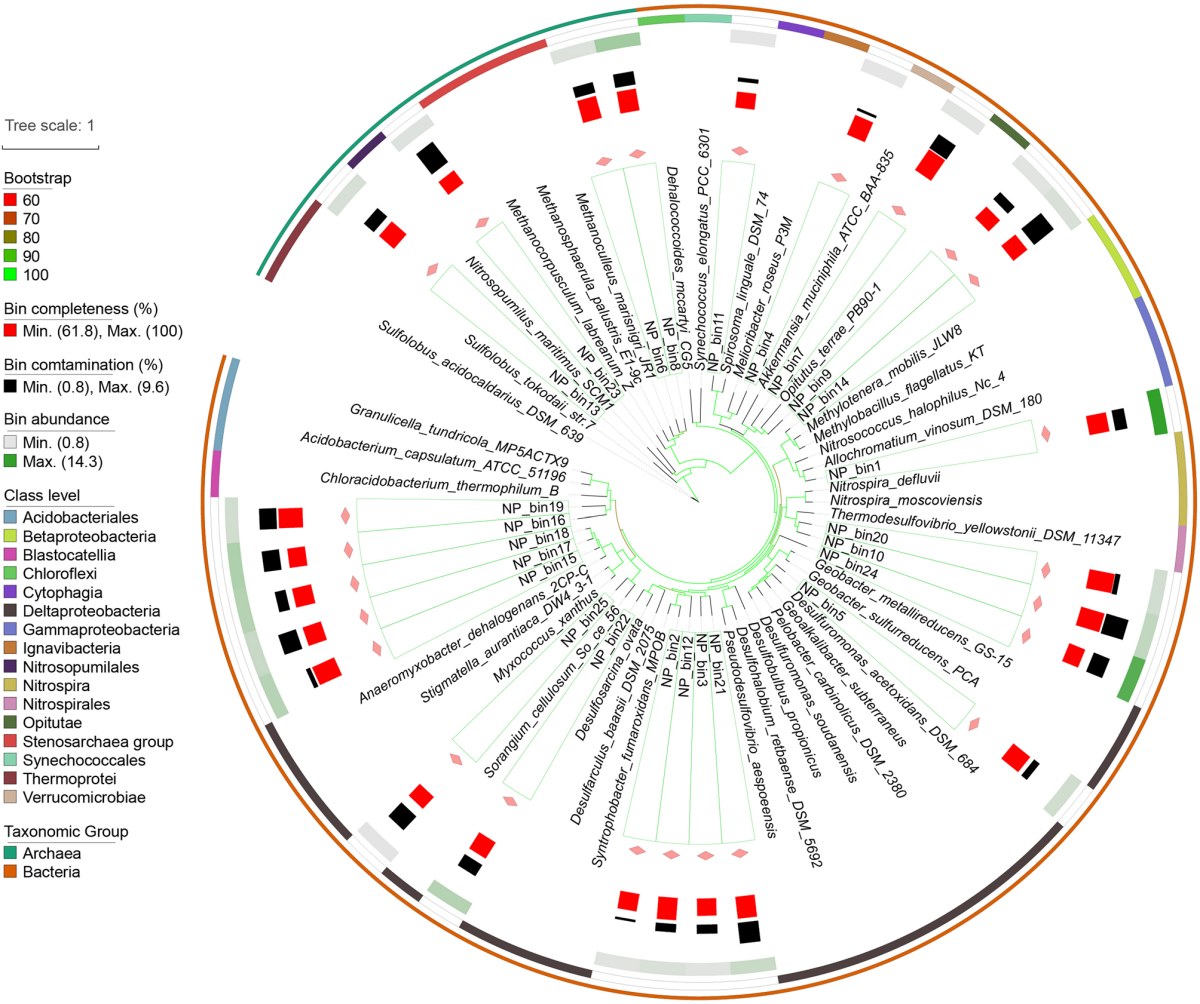
Phylogenetic characterization of 25 moderate/high-quality MAGs recovered from sediments without submerged plants. The phylogeny was generated from orthology proteins using Orthofinder software. 63 microbial genomes using IQ-TREE with a best fit LG + F + R9 model using the Bayesian Information Criterion (BIC). Bootstraps were based on 1000 replicated trees. The alignment of 63 genomes was generated using MAFFT

**Fig. 4:**
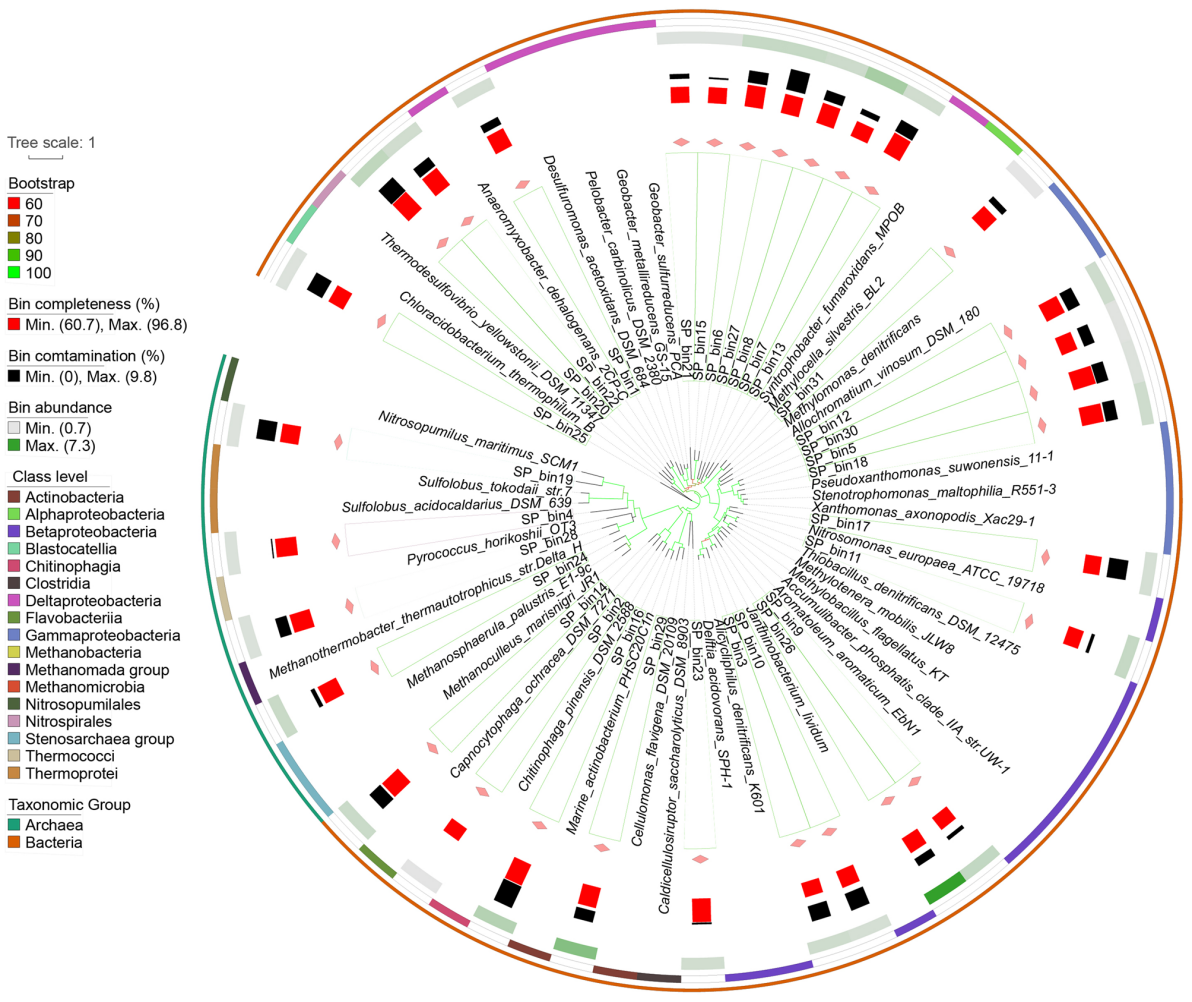
Phylogenetic characterization of 31 moderate/high-quality MAGs recovered from sediments with submerged plants. The phylogeny was generated from orthology proteins using Orthofinder software. 66 microbial genomes using IQ-TREE with a best fit LG + F + R8 model using the Bayesian Information Criterion (BIC). Bootstraps were based on 1000 replicated trees. The alignment of 66 genomes was generated using MAFFT

The 56 retrieved MAGs (25 from NP and 31 from SP) could be assigned to more than Ten phyla, including one archaeal phylum that lacks cultivated representatives (Table S1 in Supplementary file 2). For NP metagenomes, 11 bins were assigned to the class *Deltaproteobacteria*, which was identified as the dominant taxon with a total bin abundance of 27.8 (the number of genome copies per million sequences). NP_bin1 was affiliated to *Gammaproteobacteria* with a bin abundance of 14.3. Two archaeal bins (NP_bin6 and NP_8) were assigned to the class *Methanomicrobia* with a total bin abundance of 7.4. NP_bin13 was affiliated to the order *Methanomethylicales*, with bin abundance of 1.9 (Fig. 3). In the SP metagenomes, the total abundances of bins affiliated to *Betaproteobacteria* (six bins), *Deltaproteobacteria* (eight bins) and *Gammaproteobacteria* (five bins) were 15.5, 13.6 and 5.9, respectively. SP_bin26 assigned to the order *Burkholderiales* was the most abundant bin (7.3). For archaea, four bins were affilitaed to the phyla *Euryarchaeota*, *Thaumarchaeota* and *Candidatus* *Verstraetearchaeota*. Phylogenetic analysis indicated that the dominant archaeal bin (SP_bin24, bin abundance 1.8) was affiliated to the genus *Methanoculleus* (Fig. 4).

To improve understanding of the uncultured archaeal phylum *Verstraetearchaeota*, a phylogenetic analysis of a potentially divergent cluster of *mcrA* genes from these *Verstraetearchaeota* genomes was conducted (Fig. S3 in Supplementary file 2). Results showed that sequences obtained from this study clustered with natural environmental clones. For example, *mcrA* from MAG NP_bin13 was close to sequences from hot spring sediments, and *mcrA* from MAG SP_bin4 clustered with sequences from freshwater spring sediments.

## Discussion

Due to the prevalence of macrophytes in wetland ecosystems, understanding the influence of aquatic plants on the functional potential of microbial communities is becoming a hot topic in ecology and environmental science. In this study, detailed metagenomic analyses of microbial genes involved in the nitrogen cycle, sulfur cycle and methanogenesis affected by *V. natans* in urban wetlands of the Xixi National Wetland Park were performed. Our results showed that *V. natans* increased the relative abundances of most functional genes involved in the transformation of carbon, nitrogen and sulfur in the wetland sediments. Specifically, the relative abundances of functional genes involved in nitrogen fixation (*nifD/H/K/W*), assimilatory nitrate reduction (*nasA* and *nirA*), denitrification (*nirK/S* and *nosZ*), assimilatory sulfate reduction (*cysD/H/J/N/Q* and *sir*), and sulfur oxidation (*glpE, soeA, sqr* and *sseA*) in sediments with *V. natans* were all significantly higher than in unvegetated wetland sediments. Additionally, the recovered and annotated methanogen MAGs, including uncultured *Verstraetearchaeota,* indicated that hydrogenotrophic, acetoclastic, and methylotrophic methanogenesis may simultaneously contribute to methane production and therefore play important roles in carbon cycling in urban wetlands.

Submerged plants in wetland ecosystems can create aerobic microhabitats and provide rich nutrients in the rhizosphere (Srivastava et al. 2017), which facilitates the growth of specialized microorganisms and significantly affects the microbial mediation of biogeochemical cycling (Kaokniffin et al. 2010; Liu et al. 2018). We were able to annotate almost all nitrogen cycling genes in our wetland sediments, suggesting the presence of an active nitrogen cycling process in the Xixi National Wetland Park, which is consistent with a recent study (Liu et al. 2018). Moreover, many genes involved in nitrogen fixation were enriched in SP, suggesting that the potential nitrogen sink capacity of sediments was enhanced by *V. natans*. The gene abundances of *ureA/*C that catalyzes the conversion of organic nitrogen to ammonia nitrogen were significantly higher in SP than in NP sediments. The enhancement of the ammonification process is important for organic nitrogen removal and nitrogen cycling. The higher abundances of the genes *nasA* and *nirA* in SP sediments increased the transformation of NO_3_^−^ to NH_4_^+^. Also, the abundances of *nirS/K* genes were significantly higher in SP than in NP sediment, which is consistent with a previous study on oligotrophic shallow lakes (Vilacosta et al. 2016). Furthermore, the denitrification process was not limited by carbon in vegetated sediments due to plant root exudates or the decay of plant tissue, both of which provide sufficient carbon sources for microorganisms (Vilacosta et al. 2016). The abundance of the *nosZ* gene was significantly higher in the vegetated than the unvegetated sediments, indicating that submerged plants may reduce N_2_O production from wetlands. However, García-Lledó et al. (2011) showed that *Phragmites* and *Typha* have the potential for high N_2_O emissions in vegetated sediments. Therefore, it is necessary to assess the effect of vegetation type on the production of N_2_O in wetlands, and more studies are needed to understand the relationship between wetlands and greenhouse gas emissions.

Although the concentration of sulfur compounds (e.g., sulfate and sulphide) in freshwater wetlands (e.g., lakes and ponds) is generally lower than in oceans, the sulfur cycle is still active in such habitats (Holmer and Storkholm 2001). Our studies detected almost all functional genes involved in the sulfur cycle in freshwater wetland sediments, suggesting that sulfur oxidizers and sulfate reducers are widespread in freshwater habitats. Sulfide oxidation is one of the important processes in the sulfur cycle and its metabolic pathway, which involves the genes *dsrA/B/E/F/H* and *dsrC,* is considered to be the reverse of the sulfite reduction pathway (Bell et al. 2020). The *dsrD* gene detected herein can facilitate sulfite reduction (Rabus et al. 2015). The lower abundance of the *dsrM* gene (which as a similar function to *dsrD),* detected in SP sediments indicates that submerged plants may weaken the sulfite reduction function. Additionally, the genes encoding sulfite dehydrogenase (quinone) subunit (*soeA*) and sulfide-quinone oxidoreductase (*sqr*) had higher abundances in SP than NP sediments, indicating that the conversion of sulfite to sulfate and oxidation of sulfide to elemental sulfur may be enhanced in the SP sediments. Such changes in biochemical processes could be attributed to two phenomena: (i) the aerobic environment around the roots of submerged plants could inhibit the reduction of sulfite to sulfide (Allen et al. 2002); (ii) inorganic sulfides are more cytotoxic than other sulfur compounds, so submerged plants may stimulate microbes to detoxify sulfide (Marcia et al. 2009; Xia et al. 2017). Sulfur is an essential element to maintain cell viability so sulfur compounds are synthesized through assimilation (Takahashi et al. 2011). Microorganisms play key roles in promoting sulfur uptake in plants by transforming organosulfur (Kertesz and Mirleau 2004). Cordovez et al. (2018) found that the dimethyl trisulfide released by root-associated *Microbacterium* promoted *Arabidopsis* growth via modulation of sulfur metabolism. These factors may partly explain why the abundances of many genes (such as *cysD/H/J/N/Q* and *sir*) involved in assimilatory sulfate reduction were significantly higher in SP than NP sediments.

Methanogenesis is one of the important parts of the carbon cycle in wetlands, and the annual methane release from wetlands accounts for more than 20% of global methane emissions (Laanbroek 2010). We detected hydrogenotrophic, acetoclastic and methylotrophic methanogenesis in the sediments, suggesting these metabolic pathways contributed to methane release in urban wetlands. However, when methyl substances are limiting, acetoclastic methanogens usually dominate methane production in freshwater wetlands (Lyu et al. 2018). The MAGs of NP_bin6 and SP_bin28 (*Methanomicrobiales*) have all the genes necessary for the utilization of acetate, however a previous study suggested that acetoclastic methanogens were only affiliated to the *Methanosarcinales* group (Borrel et al. 2011). A possible explanation for this inconsistency is that genes regulating the acetoclastic methanogenesis from non-*Methanosarcinales* taxa are not expressed.

*Methanomicrobiales* and *Methanobacteriaceae* were the dominant methanogenic archaean lineages in the wetland sediments, which is consistent with previous sequence-based studies of amplicons in freshwater wetlands (Lin et al. 2015; Liu et al. 2012). Furthermore, SP_bin28 (*Methanobacteriaceae*) harbored a large number of electron transporters (e.g., ABC-type transporters) encoded in their genomes, which could help microbial adaptation to low substrate (H_2_) environments (Browne et al. 2017). Notably, SP_bin4 (*Methanomethylicales*) with obligate H_2_-dependent methylotrophic methanogenesis belongs to the archaeal phylum *Candidatus* *Verstraetearchaeota* (Vanwonterghem et al. 2016). The near-complete genomes of *Methanomethylicales* from the freshwater wetland sediments were analyzed in current study. These genomes harbor the genes for heterodisulfide reductase (Hdr)/[Ni-Fe] hydrogenase complex for heterodisulfide coenzyme B coenzyme M (CoB-S-SCoM) reduction and H_2_ oxidation (Zhang et al. 2020).

The abundance of *nirS/K* and *mcrA2* genes was higher in the SP than the NP sediments, suggesting a promotion of nitrite-dependent anaerobic methane oxidation (Hu et al. 2014). At the same time, the accumulation of plant-derived carbon sources may have given rise to a relatively high C:S ratio (Chen et al. 2014), which decreased the electron transfer between anaerobic methanotrophic archaea and sulfate-reducing bacteria (Wegener et al. 2015), finally resulting in poor methane oxidation. In addition, the presence of plants had different effects on the production of different greenhouse gases due to microbial processes. For example, the abundance of the *nosZ* gene, which encodes nitrous oxide reductase, increased in sediments with *V. natans* and may promote the conversion of N_2_O to N_2_. However, the abundance of *mcrA2* in SP was slightly higher than in NP, which may favor methane production. Therefore, the impacts of vegetation on greenhouse gas production should also be considered when designing wetlands for ecological restoration. From the perspective of environmental protection, molecular markers, such as microbial functional genes, could provide useful information for improving the ecological evaluation of wetland ecosystems.

In summary, sedimental microorganisms from the Xixi National Wetland Park wetlands have strong biogeochemical potential, including microbially mediated methane, nitrogen and sulfur cycling. Vegetation significantly increases the abundance of functional genes involved in sedimental nitrogen fixation, assimilatory nitrate reduction, denitrification, assimilatory sulfate reduction, and sulfur oxidation. Moreover, we found a suitable C:N ratio was important for maintaining stable microbial community structure and function in wetland ecosystems. Additionally, hydrogenotrophic, acetoclastic, and methylotrophic methanogenesis may contribute significantly to the production of methane in wetland sediments. This study expands our understanding of functional diversity of urban wetland microbial communities and the effects of aquatic plants on them, which provides a basis for further mining of wetland microbial "dark matter".

## Materials and methods

### Study area and sampling procedures

All samples involved in this study were collected from the Xixi National Wetland Park (30°15′ ~ 30°17′ N and 120°1′ ~ 120°6′ E) in Hangzhou, China, which is the oldest urban wetland park in China and has a total area of 11.5 km^2^. The vegetation not only enhances the scenic amenity but also provides water purification functions in the wetlands. So, this is an excellent natural laboratory to study changes and adaptations of microbial functions in biogeochemical cycling. To reveal the effects of submerged plants on microbial functional genes and the corresponding changes in wetland ecosystem functioning, two types of sediment were investigated: five sites with submerged plants, primarily *V. natans* (SP) and five sites with no plants (NP). The upper layer of sediments (0–10 cm) was collected using a Peterson grab sampler in May 2018. All the collected samples were transported in a cooler box to the laboratory for further analysis.

### Chemical measurements

After drying the sediments at 30 °C until they reached a constant weight, chemical characteristics including total nitrogen (TN) and total carbon (TC) were determined with an elemental analyzer (EA3000, EuroVector, German). Total phosphorus (TP) was measured by an inductively coupled plasma spectrometry (ICP) instrument (Prodigy7, Leeman Labs, USA). Conductivity and pH were determined as previously described (Wang et al. 2020). All samples were analyzed in three replicates (Table S2 in Supplementary file 2).

### DNA extraction and purification

After removing impurities (e.g., plant roots and grit) from the sediment samples, microbial community DNA was extracted using a freeze-grinding plus sodium dodecyl sulfate (SDS) lysis method as described previously (Zhou et al. 1996), followed by purification using the PowerSoil DNA Extraction kit (MoBio Laboratories, CA, USA) according to manufacturer’s protocol. The extracted DNA was dissolved in 50 μl elution buffer. DNA concentrations were determined using a NanoDrop-2000 fluorospectrometer (Thermo Fisher Scientific, MA, USA). All DNA samples were stored at − 80 °C until further processing.

### Shot-gun metagenome sequencing and analysis

For each sample, 1 μg of genomic DNA was used for library construction using VAHTSTM Universal DNA library Prep Kit for Illimina® V3 (ND607) and then sequenced on an Illumina NextSeq 550 platform (paired-end 150 bp). A total of 265.4 Gb raw reads generated for ten samples were qualified using the Read_qc module in metaWRAP, which is a flexible pipeline for genome-resolved metagenomic data analysis (Uritskiy et al. 2018). Only good quality reads (197.9 Gb) were used for downstream analysis (Supplementary file 3). These were annotated with biological functions (i.e., methane, nitrogen and sulfur cycling) using the Diamond tool (Buchfink et al. 2015) in Kyoto Encyclopedia of Genes and Genomes (KEGG), NCycDB (Tu et al. 2019) and ScycDB (Yu et al. 2021). Reads targeted by databases were extracted and used to build a relative abundance functional profile. A function for random subsampling each sample at the lowest sequencing depth was included (Tu et al. 2019).

### Assembly, binning and annotation

The resulting sequences were put into metaWRAP for metagenomic assembly and binning. Briefly, the qualified reads were assembled using MEGAHIT with default parameters (Li et al. 2016). The resulting contigs are listed in Table S4 in Supplementary file 2. Binning of the assembled metagenome was computed and refined using MaxBin2 (Wu et al. 2016) and metaBAT2 (Kang et al. 2015). Levels of completion and contamination of all the bins were evaluated with CheckM (Parks et al. 2015). Bins with completion of > 60% and contamination of < 10% were subjected to annotation of taxonomy using Taxator-tk (Chaumeil et al. 2019). The abundance of bins in each sample was estimated, and the Salmon software was used to quantify individual contigs (Patro et al. 2017). Gene functions of those bins assigned to archaea were annotated with methanogenesis using the KEGG server (BlastKOALA) (Kanehisa et al. 2016) and eggNOG-mapper (Huertacepas et al. 2017). The annotated information is listed in Supplementary file 5.

### Phylogenetic analyses

The phylogenetic tree of MAGs was constructed using OrthoFinder algorithm software (Emms and Kelly 2015) and IQ-TREE (Nguyen et al. 2015). Briefly, reference genomes were downloaded from the NCBI (https://www.ncbi.nlm.nih.gov) database. Each gene at the amino acid sequence level was aligned using the MAFFT software integrated into OrthoFinder (Katoh and Standley 2014) and the phylogenomic tree was generated using IQ-TREE with a best-fit model.

The methyl coenzyme M reductase alpha subunit encoded by *mcrA* catalyzes methanogenesis, so the *mcrA* gene was used as a marker gene for detecting methanogens (Evans et al. 2015). The phylogeny of *mcrA* was used to identify the placement of the *Verstraetearchaeota mcrA*. The retrieved *mcrA* sequences were aligned using ClustalW (Larkin et al. 2007) with all *Verstraetearchaeota mcrA* sequences downloaded from the NCBI and IMG-M (https://img.jgi.doe.gov/cgi-bin/m/main.cgi) databases on August 3, 2020. A phylogenetic tree for the *mcrA* gene was generated using IQ-tree with parameters: iqtree -m TIM2 + F + R5 -bb 1000 -redo -alrt 1000. The phylogenomic trees were constructed using iTOL (Letunic and Bork 2016).

## Supplementary Information

Below is the link to the electronic supplementary material.

Supplementary file1 (DOCX 32 KB)

Supplementary file2 (DOCX 487 KB)

Supplementary file3 (DOCX 51 KB)

Supplementary file4 (DOCX 24 KB)

Supplementary file5 (XLSX 28 KB)

## Data Availability

All the raw DNA sequences (metagenomic reads) are available in the Genome Sequence Archive (GSA) under project PRJCA003534, publicly accessible at https://bigd.big.ac.cn/gsa/.
