# Supplementary material for: Metagenomic insights into the effects of submerged plants on functional potential of microbial communities in wetland sediments
Source: Mar Life Sci Technol. 2021 Aug 27;3(4):405–15. doi: 10.1007/s42995-021-00100-3 (PMC10077182; doi:10.1007/s42995-021-00100-3)
Supplement: Supplementary file 1 — Supplementary file1 (DOCX 32 KB) [file 42995_2021_100_MOESM1_ESM.docx]

Summary of representative sequences for selected gene families in NcycDB

| Pathway | Gene（sub）family | Annotation | Representative sequences | | |
| --- | --- | --- | --- | --- | --- |
|  |  |  | No plants | Submerged plants | *p* |
| Nitrification | *amoA*_A | ammonia monooxygenase subunit A (archaea) | / | 2 (2) |  |
|  | *amoB*_A | ammonia monooxygenase subunit B (archaea) | 3 (2) | 2 (1) | 0.553 |
|  | *amoC*_A | ammonia monooxygenase subunit C (archaea) | / | 2 (1) |  |
|  | *amoA*_B | ammonia monooxygenase subunit A (bacteria) | / | 1 (1) |  |
|  | *amoB*_B | ammonia monooxygenase subunit B (bacteria) | / | 1 (1) |  |
|  | *amoC*_B | ammonia monooxygenase subunit C (bacteria) | 1 (1) | / |  |
|  | *hao* | hydroxylamine dehydrogenase | 319 (21) | 239 (33) | **0.008** |
|  | *nxrA* | nitrite oxidoreductase, alpha subunit | 8 (4) | 7 (3) | 0.569 |
|  | *nxrB* | nitrite oxidoreductase, beta subunit | 21 (5) | 15 (7) | 0.168 |
| Denitrification | *napA* | periplasmic nitrate reductase NapA | 1287 (61) | 1319 (83) | 0.201 |
|  | *napB* | cytochrome c-type protein NapB | 29 (5) | 37 (11) | 0.302 |
|  | *napC* | cytochrome c-type protein NapC | 54 (16) | 110 (21) | **0.021** |
|  | *narG* | nitrate reductase | 777 (71) | 666 (59) | 0.028 |
|  | *narH* | nitrate reductase | 373 (24) | 336 (45) | 0.101 |
|  | *narI* | nitrate reductase gamma subunit | 153 (25) | 154 (17) | 0.935 |
|  | *narJ* | nitrate reductase molybdenum cofactor assembly chaperone | 128 (25) | 172 (17) | **0.001** |
|  | *nirK* | nitrite reductase (NO-forming) | 242 (35) | 388 (65) | **0.004** |
|  | *nirS* | nitrite reductase (NO-forming) | 425 (20) | 631 (66) | **0.004** |
|  | *norB* | nitric oxide reductase subunit B | 722.8 (15) | 649.8 (15) | **0.004** |
|  | *norC* | nitric oxide reductase subunit C | 76 (4) | 77 (11) | 0.921 |
|  | *nosZ* | nitrous-oxide reductase | 469 (52) | 630 (34) | **0.011** |
|  | *narZ* | nitrate reductase 2, alpha subunit | 390 (46) | 477 (45) | **0.001** |
|  | *narY* | nitrate reductase 2, beta subunit | 26 (4) | 66 (12) | **0.003** |
|  | *narV* | nitrate reductase 2, gamma subunit | 2 (1) | 7 (3) | **0.025** |
|  | *narW* | nitrate reductase 2, delta subunit | 4 (1) | 1 (1) | **0.033** |
| Assimilatory nitrate reduction | *nasA* | assimilatory nitrate reductase catalytic subunit | 1018 (32) | 1172 (49) | **0.002** |
|  | *nasB* | assimilatory nitrate reductase electron transfer subunit | 64 (10) | 72 (12) | 0.171 |
|  | *nirA* | ferredoxin-nitrite reductase | 155 (29) | 253 (34) | **0.001** |
|  | *NR* | nitrate reductase (NAD(P)H) | 252 (15) | 299 (37) | **0.032** |
|  | *narB* | assimilatory nitrate reductase | 532 (22) | 567 (36) | **0.013** |
|  | *narC* | cytochrome b-561 | 285 (28) | 307 (21) | **0.023** |
| Dissimilatory nitrate reduction | *napA* | periplasmic nitrate reductase NapA | 1287 (61) | 1319 (83) | 0.201 |
|  | *napB* | cytochrome c-type protein NapB | 29 (5) | 37 (11) | 0.302 |
|  | *napC* | cytochrome c-type protein NapC | 54 (16) | 110 (21) | **0.021** |
|  | *narG* | nitrate reductase | 777 (71) | 666 (59) | **0.028** |
|  | *narH* | nitrate reductase | 373 (24) | 336 (45) | 0.101 |
|  | *narI* | nitrate reductase gamma subunit | 153 (25) | 154 (17) | 0.935 |
|  | *narJ* | nitrate reductase molybdenum cofactor assembly chaperone | 128 (25) | 172 (17) | **0.001** |
|  | *narZ* | nitrate reductase 2, alpha subunit | 390 (46) | 477 (45) | **0.001** |
|  | *narY* | nitrate reductase 2, beta subunit | 26 (4) | 66 (12) | **0.003** |
|  | *narV* | nitrate reductase 2, gamma subunit | 2 (1) | 7 (3) | **0.025** |
|  | *narW* | nitrate reductase 2, delta subunit | 4 (1) | 1 (1) | **0.033** |
|  | *nirB* | nitrite reductase (NADH) large subunit | 596 (32) | 744 (57) | **0.010** |
|  | *nirD* | nitrite reductase (NADH) small subunit | 181 (22) | 248 (35) | **0.014** |
|  | *nrfA* | nitrite reductase (cytochrome c-552) | 392 (19) | 400 (30) | 0.639 |
|  | *nrfB* | cytochrome c-type protein NrfB | 3 (1) | 1 (1) | 0.120 |
|  | *nrfC* | protein NrfC | 2107 (65) | 1851 (96) | **0.023** |
|  | *nrfD* | protein NrfD | 50 (9) | 40 (8) | 0.139 |
| Nitrogen fixation | *anfG* | nitrogenase delta subunit | / | / |  |
|  | *nifD* | nitrogenase molybdenum-iron protein alpha chain | 97 (16) | 148 (17) | **0.013** |
|  | *nifH* | nitrogenase iron protein NifH | 107 (18) | 153 (16) | **0.008** |
|  | *nifK* | nitrogenase molybdenum-iron protein beta chain | 77 (27) | 131 (20) | **0.032** |
|  | *nifW* | nitrogenase-stabilizing/protective protein | 3 (2) | 7 (1) | **0.006** |
| Anammox | *hzo* | hydrazine oxidoreductase | 5 (2) | 3 (2) | **0.047** |
|  | *hzsA* | hydrazine synthase subunit A | 9 (3) | 11 (4) | 0.313 |
|  | *hzsB* | hydrazine synthase subunit B | 5 (1) | 5 (2) | 1.000 |
|  | *hzsC* | hydrazine synthase subunit C | 1 (1) | / |  |
|  | *hdh* | hydrazine dehydrogenase | 1 (1) | / |  |
| Organic degradation and synthesis | *ureA* | urease subunit gamma | 39 (5) | 58 (9) | **0.001** |
|  | *ureB* | urease subunit beta | 36 (5) | 39 (3) | **0.021** |
|  | *ureC* | urease subunit alpha | 221 (40) | 333 (30) | **0.001** |
|  | *nao* | nitroalkane oxidase | 2 (1) | 6 (5) | 0.119 |
|  | *nmo* | nitronate monooxygenase | 2704 (130) | 2717 (92) | 0.866 |
|  | *gdh*_K00260 | glutamate dehydrogenase | 51 (6) | 47 (6) | 0.434 |
|  | *gdh*_K00261 | glutamate dehydrogenase (NAD(P)+) | 1098 (48) | 1046 (66) | 0.325 |
|  | *gdh*_K00262 | glutamate dehydrogenase (NADP+) | 604.4 (34) | 570 (47) | 0.340 |
|  | *gdh*_K15371 | glutamate dehydrogenase | 1073 (197) | 1312 (157) | **0.009** |
|  | *gs*_K00264 | glutamate synthase (NADPH/NADH) | 50 (9) | 57 (6) | 0.309 |
|  | *gltB* | glutamate synthase (NADPH/NADH) large chain | 1567 (92) | 1939 (73) | **0.001** |
|  | *gltD* | glutamate synthase (NADPH/NADH) small chain | 9410 (294) | 8692 (454) | **0.032** |
|  | *gs*_K00284 | glutamate synthase (ferredoxin) | 883 (98) | 896 (122) | 0.844 |
|  | *glsA* | glutaminase | 704 (101) | 804 (91) | 0.143 |
|  | *glnA* | glutamine synthetase | 3159 (65) | 3474 (118) | **0.001** |
|  | *asnB* | asparagine synthase (glutamine-hydrolysing) | 3320 (57) | 3464 (60) | **0.027** |
|  | *ansB* | glutamin-(asparagin-)ase | 493 (60) | 430 (36) | 0.185 |
| Others | *hcp* | hydroxylamine reductase | 448 (65) | 460 (64) | 0.538 |
|  | *pmoA* | particulate methane monooxygenase subunit A | 7 (2) | 11 (5) | 0.054 |
|  | *pmoB* | particulate methane monooxygenase subunit B | 18 (3) | 24 (4) | 0.062 |
|  | *pmoC* | particulate methane monooxygenase subunit C | 13 (2) | 15 (4) | 0.531 |

Data are presented as mean (Standard error); /: either not detected in our data, or the average number of sequences was less than 1. *p* value was obtained by two-sample Student's t test
