# Supplementary material for: Metagenomic insights into the effects of submerged plants on functional potential of microbial communities in wetland sediments
Source: Mar Life Sci Technol. 2021 Aug 27;3(4):405–15. doi: 10.1007/s42995-021-00100-3 (PMC10077182; doi:10.1007/s42995-021-00100-3)
Supplement: Supplementary file 2 — Supplementary file2 (DOCX 487 KB) [file 42995_2021_100_MOESM2_ESM.docx]

**Supplementary information**

**Metagenomic insights into the effects of submerged plants on functional potential of microbial communities in wetland sediments**

Binhao Wang^a,b^, Xiafei Zheng^a^, Hangjun Zhang^c^, Xiaoli Yu^a^, Yingli Lian^a^, Xueqin Yang^a^, Huang Yu^a^, Ruiwen Hu^a^, Zhili He^a,d^, Fanshu Xiao^a,^*, Qingyun Yan^a^^,^*

^a^ *Environmental Microbiomics Research Center, School of Environmental Science and Engineering, Southern Marine Science and Engineering Guangdong Laboratory (Zhuhai), Sun Yat-sen University, Guangzhou 510006, China*

^b^ *Institute of Soil and Water Resources and Environmental Science, College of Environmental and Resource Sciences, Zhejiang University, Hangzhou 310058, China*

^c^ *College of Life and Environmental Sciences, Hangzhou Normal University, Hangzhou 310036, China*

^d^ *College of Agronomy, Hunan Agricultural University, Changsha 410128, China*

* Corresponding authors.

E-mail address: [yanqy6@mail.sysu.edu.cn](mailto:yanqy6@mail.sysu.edu.cn) (Q. Yan), [xiaofansh@mail.sysu.edu.cn](mailto:xiaofansh@mail.sysu.edu.cn) (F. Xiao).

The following supplementary information (SI) includes 4 tables and 3 figures.

**1. SUPPLEMENTARY TABLES**

**Table S1**

Features of all genome bins > 60% completeness and with < 10% contamination.

| ID | Taxon | Number  Scaffolds | Genome Size  (bp) | GC  Content (%) | N50 value  (bp) | Number  CDS | Number  rRNA | Number  tRNA | Completeness (%) | Contamination (%) |
| --- | --- | --- | --- | --- | --- | --- | --- | --- | --- | --- |
| NP_bin1 | Gammaproteobacteria | 587 | 2694073 | 69.2 | 5594 | 2655 | 1 | 41 | 78.47 | 4.559 |
| NP_bin2 | Deltaproteobacteria | 635 | 2535339 | 56.6 | 4582 | 2564 | 0 | 19 | 68.73 | 0.842 |
| NP_bin3 | Deltaproteobacteria | 708 | 2117133 | 58.8 | 3180 | 2001 | 0 | 13 | 67.48 | 3.387 |
| NP_bin4 | Bacteria | 714 | 2853900 | 43.1 | 4526 | 2485 | 0 | 29 | 82.76 | 0.928 |
| NP_bin5 | Desulfuromonadales | 188 | 1902609 | 62.8 | 17558 | 1797 | 2 | 26 | 83.88 | 2.580 |
| NP_bin6 | Methanomicrobiaceae | 436 | 1406389 | 65.0 | 3235 | 1401 | 0 | 35 | 88.74 | 3.995 |
| NP_bin7 | Verrucomicrobiae | 562 | 3356143 | 60.3 | 8413 | 3086 | 1 | 40 | 96.84 | 6.245 |
| NP_bin8 | Methanomicrobiaceae | 137 | 1272768 | 53.5 | 6035 | 1483 | 1 | 37 | 85.73 | 5.000 |
| NP_bin9 | Bacteria | 614 | 2110948 | 59.5 | 3839 | 2188 | 0 | 33 | 61.80 | 3.603 |
| NP_bin10 | Thermodesulfovibrionales | 209 | 2184520 | 48.2 | 16764 | 2131 | 0 | 46 | 94.54 | 8.545 |
| NP_bin11 | Cytophagales | 543 | 1776324 | 45.1 | 3531 | 1749 | 0 | 17 | 62.11 | 1.488 |
| NP_bin12 | Deltaproteobacteria | 657 | 3236187 | 60.2 | 6581 | 3061 | 2 | 36 | 87.14 | 3.123 |
| NP_bin13 | Methanomethylicales | 128 | 969174 | 51.2 | 6285 | 1087 | 1 | 22 | 76.32 | 4.828 |
| NP_bin14 | Betaproteobacteria | 506 | 1658641 | 57.6 | 3614 | 1840 | 1 | 32 | 69.74 | 9.422 |
| NP_bin15 | Anaeromyxobacteraceae | 298 | 3525779 | 73.0 | 18257 | 3304 | 0 | 36 | 93.78 | 1.612 |
| NP_bin16 | Bacteria | 762 | 2488489 | 72.4 | 3472 | 2274 | 0 | 33 | 69.08 | 6.475 |
| NP_bin17 | Anaeromyxobacteraceae | 449 | 2470367 | 72.2 | 7162 | 2418 | 0 | 29 | 74.47 | 6.935 |
| NP_bin18 | Anaeromyxobacteraceae | 1099 | 3051637 | 73.8 | 2912 | 3024 | 1 | 43 | 75.81 | 4.055 |
| NP_bin19 | Bacteria | 190 | 3685458 | 66.7 | 41155 | 3114 | 1 | 44 | 93.82 | 6.410 |
| NP_bin20 | Thermodesulfovibrionales | 380 | 2873714 | 57.8 | 11415 | 2889 | 2 | 37 | 100 | 1.868 |
| NP_bin21 | Deltaproteobacteria | 467 | 2358908 | 58.7 | 6457 | 2362 | 1 | 32 | 85.80 | 7.790 |
| NP_bin22 | Deltaproteobacteria | 751 | 3380256 | 60.3 | 5377 | 3222 | 0 | 30 | 73.82 | 4.888 |
| NP_bin23 | Archaea | 763 | 1487954 | 46.4 | 1497 | 1428 | 1 | 4 | 62.62 | 9.596 |
| NP_bin24 | Bacteria | 761 | 1262245 | 68.9 | 1657 | 1713 | 0 | 20 | 66.31 | 6.401 |
| NP_bin25 | Myxococcales | 1508 | 5709389 | 72.6 | 4972 | 5746 | 0 | 40 | 62.45 | 7.017 |
| SP_bin1 | Anaeromyxobacteraceae | 501 | 2875469 | 73.2 | 7054 | 2791 | 1 | 31 | 81.03 | 3.369 |
| SP_bin2 | Bacteroidetes | 693 | 2458046 | 41.7 | 4020 | 2254 | 1 | 20 | 61.20 | 0 |
| SP_bin3 | Burkholderiales | 932 | 3077541 | 69.2 | 3631 | 3170 | 1 | 24 | 60.72 | 6.546 |
| SP_bin4 | Methanomethylicales | 205 | 996923 | 51.8 | 5941 | 1184 | 2 | 22 | 86.55 | 0.467 |
| SP_bin5 | Xanthomonadaceae | 235 | 2129230 | 59.2 | 14567 | 2094 | 2 | 39 | 94.96 | 4.977 |
| SP_bin6 | Deltaproteobacteria | 218 | 2764647 | 46.7 | 10779 | 2733 | 0 | 41 | 88.98 | 5.002 |
| SP_bin7 | Deltaproteobacteria | 396 | 1374175 | 58.8 | 3903 | 1402 | 1 | 23 | 66.24 | 2.311 |
| SP_bin8 | Deltaproteobacteria | 770 | 3737684 | 58.2 | 5851 | 3422 | 2 | 29 | 86.55 | 4.236 |
| SP_bin9 | Rhodocyclaceae | 159 | 1838417 | 65.5 | 16619 | 1834 | 0 | 29 | 63.14 | 1.594 |
| SP_bin10 | Oxalobacteraceae | 1026 | 3186124 | 65.3 | 3424 | 3202 | 0 | 32 | 72.75 | 7.524 |
| SP_bin11 | Betaproteobacteria | 212 | 1630764 | 63.3 | 10486 | 1707 | 0 | 28 | 64.40 | 1.315 |
| SP_bin12 | Gammaproteobacteria | 575 | 2458644 | 64.6 | 5090 | 2379 | 0 | 30 | 82.98 | 5.363 |
| SP_bin13 | Deltaproteobacteria | 595 | 3308867 | 61.0 | 7553 | 3180 | 0 | 30 | 92.09 | 5.326 |
| SP_bin14 | Flavobacteriaceae | 339 | 2424643 | 40.8 | 5506 | 2165 | 6 | 41 | 89.37 | 6.066 |
| SP_bin15 | Deltaproteobacteria | 456 | 1718059 | 52.6 | 4225 | 1838 | 0 | 29 | 65.39 | 0.668 |
| SP_bin16 | Agromyces | 741 | 3977330 | 71.9 | 6634 | 3888 | 1 | 53 | 94.64 | 9.764 |
| SP_bin17 | Betaproteobacteria | 463 | 1772666 | 59.4 | 4528 | 1911 | 0 | 24 | 67.03 | 7.925 |
| SP_bin18 | Xanthomonadaceae | 456 | 2573318 | 67.4 | 7045 | 2532 | 1 | 42 | 91.16 | 5.052 |
| SP_bin19 | Thaumarchaeota | 454 | 1448327 | 31.2 | 2643 | 1460 | 3 | 15 | 76.10 | 7.667 |
| SP_bin20 | Thermodesulfovibrionales | 471 | 2948715 | 57.8 | 12330 | 3038 | 5 | 69 | 95.45 | 7.777 |
| SP_bin21 | Deltaproteobacteria | 452 | 1977553 | 59.5 | 5058 | 2025 | 1 | 19 | 65.55 | 2.007 |
| SP_bin22 | Bacteria | 218 | 2151476 | 48.4 | 16728 | 2147 | 0 | 43 | 89.09 | 4.545 |
| SP_bin23 | Clostridiales | 277 | 3204504 | 40.7 | 20385 | 2964 | 4 | 22 | 96.8 | 0.709 |
| SP_bin24 | Methanomicrobiaceae | 444 | 1391257 | 65.1 | 3519 | 1552 | 0 | 30 | 84.37 | 1.681 |
| SP_bin25 | Bacteria | 879 | 2488140 | 67.6 | 2957 | 2487 | 0 | 24 | 69.08 | 7.211 |
| SP_bin26 | Burkholderiales | 676 | 2194124 | 70.9 | 3599 | 2269 | 0 | 27 | 70.77 | 3.415 |
| SP_bin27 | Deltaproteobacteria | 679 | 3260476 | 45.6 | 4449 | 3020 | 0 | 54 | 80.00 | 8.668 |
| SP_bin28 | Methanobacteriaceae | 174 | 1554405 | 44.0 | 7717 | 1717 | 2 | 36 | 85.59 | 4.800 |
| SP_bin29 | Intrasporangiaceae | 993 | 3130211 | 71.0 | 3391 | 3136 | 2 | 37 | 85.05 | 4.702 |
| SP_bin30 | Xanthomonadaceae | 438 | 1664083 | 61.9 | 5440 | 1688 | 0 | 18 | 68.87 | 4.612 |
| SP_bin31 | Methylocystaceae | 588 | 1355148 | 49.6 | 2479 | 1311 | 3 | 36 | 70.51 | 2.630 |

NP: No plants; SP: Submerged plants

**Table S2**

The physicochemical parameters of sediments.

| Sediment type | pH | Conductivity  (μs/cm) | TP  (g/kg) | TN  (g/kg) | TC  (g/kg) | C/N |
| --- | --- | --- | --- | --- | --- | --- |
| No Plants | 5.94±0.10^b^ | 94.2±3.19^b^ | 0.88±0.01^b^ | 2.76±0.26^b^ | 19.52±0.55^b^ | 7.12±0.51^b^ |
| Submerged plants | 7.44±0.18^a^ | 220.6±5.69^a^ | 0.99±0.05^a^ | 4.08±0.25^a^ | 34.1±1.38^a^ | 8.37±0.24^a^ |

Values are means ± standard deviation; the different letter means the statistical difference between watershed types (*p* < 0.05). TP: Total phosphorus; TC: Total carbon; TN: Total nitrogen; C/N: Total carbon/Total nitrogen.

**Table S3**

Summary of metagenomic sequencing data for each sample.

| Sample | Raw reads (GB) | Clean reads (GB) | Raw/Clean (%) |
| --- | --- | --- | --- |
| NP1_1 | 13.38 | 10.41 | 77.76 |
| NP1_2 | 13.38 | 9.48 | 70.85 |
| NP2_1 | 15.71 | 12.27 | 78.11 |
| NP2_2 | 15.71 | 11.14 | 70.92 |
| NP3_1 | 10.52 | 7.84 | 74.53 |
| NP3_2 | 10.52 | 7.01 | 66.69 |
| NP4_1 | 11.42 | 9.04 | 79.16 |
| NP4_2 | 11.42 | 8.22 | 72.02 |
| NP5_1 | 11.95 | 9.56 | 79.98 |
| NP5_2 | 11.95 | 8.73 | 73.08 |
| SP1_1 | 16.50 | 13.08 | 79.25 |
| SP1_2 | 16.50 | 11.92 | 72.25 |
| SP2_1 | 13.56 | 10.68 | 78.72 |
| SP2_2 | 13.56 | 9.70 | 71.50 |
| SP3_1 | 13.43 | 10.56 | 78.62 |
| SP3_2 | 13.43 | 9.58 | 71.33 |
| SP4_1 | 12.65 | 9.85 | 77.89 |
| SP4_2 | 12.65 | 8.90 | 70.39 |
| SP5_1 | 13.62 | 10.48 | 76.98 |
| SP5_2 | 13.62 | 9.46 | 69.51 |

**Table S4**

Statistical analysis of the assembly results. Sequence data were merged from parallel samples for each group.

| Pond type | Sample | Contig Number | Assembly Length | N50 (bp) | N75 (bp) | Max Contig  (bp) | Mapping rate (%) |
| --- | --- | --- | --- | --- | --- | --- | --- |
| No plant | NP1-5 | 590,426 | 1,149,324,301 | 1,971 | 1,342 | 151,648 | 31.12 |
| Submerged  plant | SP1-5 | 602,083 | 1,182,661,409 | 1,995 | 1,345 | 85,414 | 30.80 |

**2. SUPPLEMENTARY FIGURES**


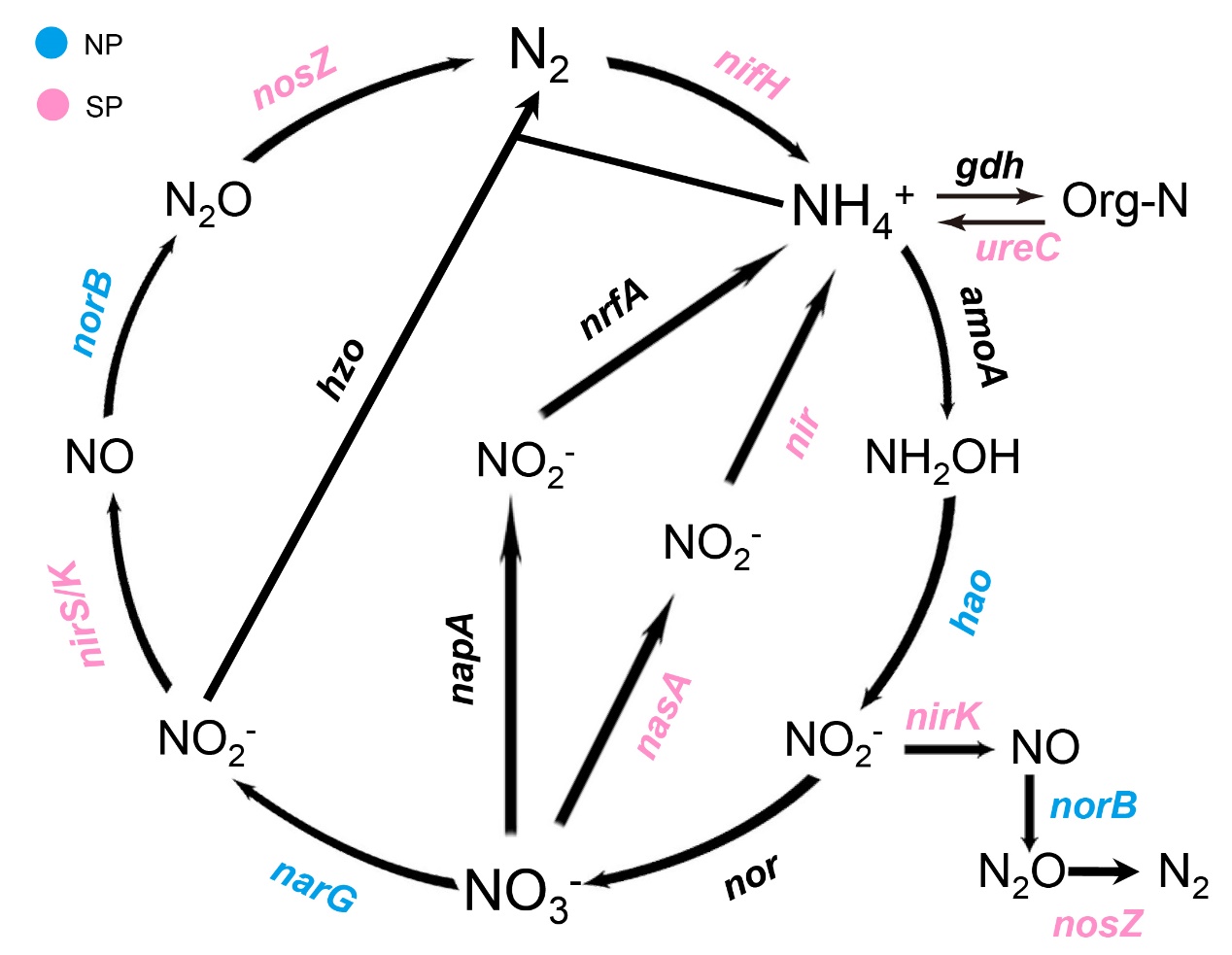


**Fig. S1** Relative changes of nitrogen-cycling genes in sediments from two watershed types. Genes with significantly increased abundance are marked in color. Blue represents significantly higher gene abundance in NP than in SP samples, whereas pink represents significantly higher gene abundance in SP samples. NP: No plants; SP: Submerged plants.


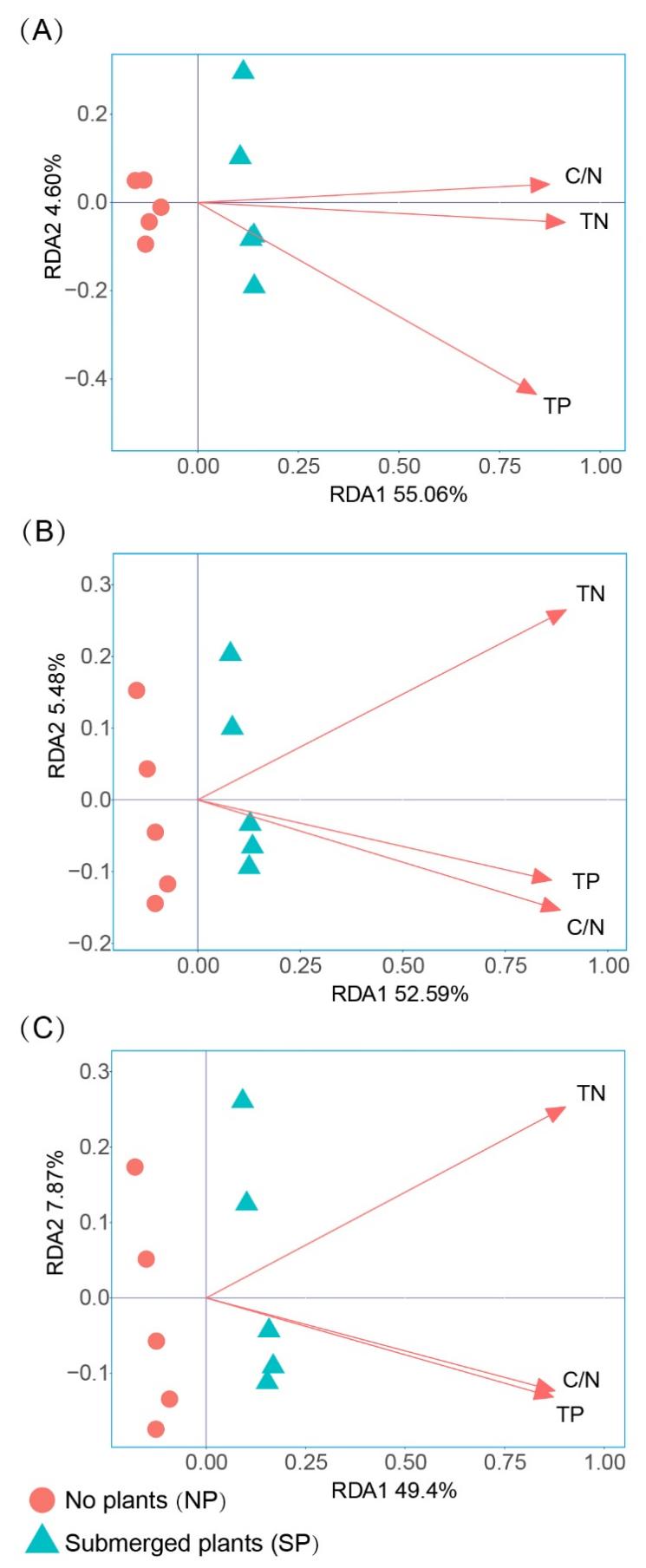


**Fig. S2** Redundancy analysis (RDA) of environmental drivers of functional genes involved in the nitrogen-cycling (A), sulfur-cycling (B) and methanogenesis (C). The values of RDA1 and RDA2 labels are percentages of variations of functional genes that they could explain. TN: Total nitrogen; TP: Total phosphorus; C/N: Total carbon/Total nitrogen.


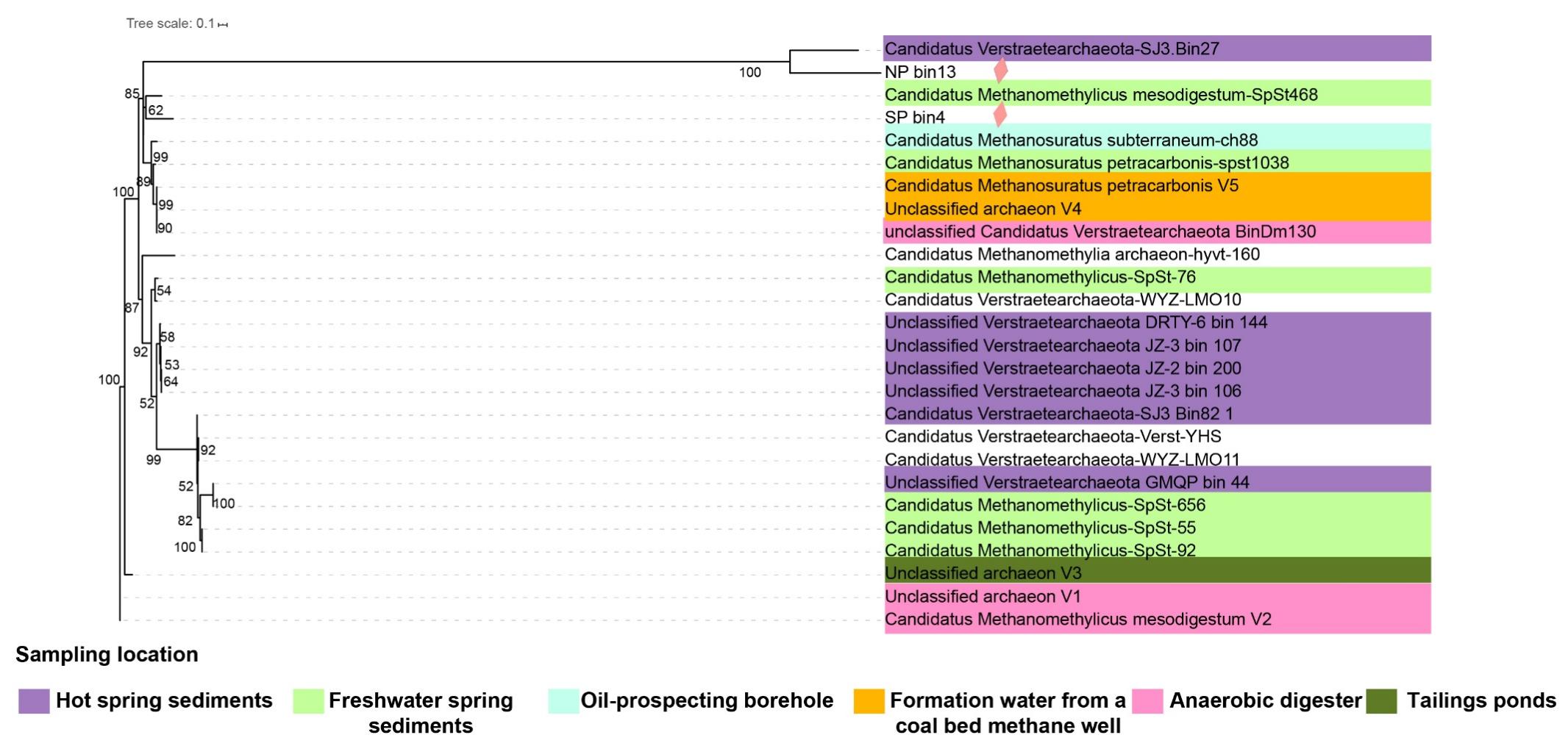


**Fig. S3** Phylogenetic trees of recovered MAGs *mcrA* gene showing the placement of the divergent *mcrA* sequences from NP_bin13 and SP_bin4.
