# Supplementary material for: Metagenomic insights into the effects of submerged plants on functional potential of microbial communities in wetland sediments
Source: Mar Life Sci Technol. 2021 Aug 27;3(4):405–15. doi: 10.1007/s42995-021-00100-3 (PMC10077182; doi:10.1007/s42995-021-00100-3)
Supplement: Supplementary file 3 — Supplementary file3 (DOCX 51 KB) [file 42995_2021_100_MOESM3_ESM.docx]

Summary of representative sequences for selected gene families in ScycDB.

| Pathways | Gene | Annotation | Representative sequences | | |
| --- | --- | --- | --- | --- | --- |
|  |  |  | No plants | Submerged plants | *p* |
| Assimilatory sulfate reduction | *cysC* | Adenylylsulfate kinase | 3981 (208) | 4515 (133) | **0.002** |
|  | *cysD* | Sulfate adenylyltransferase subunit 2 | 1030 (48) | 1293 (51) | **0.002** |
|  | *cysH* | Phosphoadenosine phosphosulfate reductase | 391 (24) | 544 (46) | **0.000** |
|  | *cysI* | Sulfite reductase (NADPH) hemoprotein beta-component | 1012 (49) | 1107 (66) | **0.025** |
|  | *cysJ* | Sulfite reductase (NADPH) flavoprotein alpha-component | 1869 (95) | 2133 (68) | **0.000** |
|  | *cysN* | Sulfate adenylyltransferase subunit 1 | 743 (31) | 945 (49) | **0.002** |
|  | *cysNC* | Bifunctional enzyme CysN/CysC | 1327 (97) | 1478 (70) | **0.040** |
|  | *cysQ* | 3'(2'), 5'-bisphosphate nucleotidase | 829 (57) | 1006 (49) | **0.004** |
|  | *nrnA* | Bifunctional oligoribonuclease and PAP phosphatase NrnA | 947 (45) | 984 (32) | 0.134 |
|  | *sat* | Sulfate adenylyltransferase | 1284 (39) | 1363 (34) | **0.013** |
|  | *sir* | Sulfite reductase (ferredoxin) | 913 (111) | 1183 (83) | **0.001** |
| Dissimilatory sulfate reduction and oxidation | *aprA* | Adenylylsulfate reductase, subunit A | 814 (33) | 848 (40) | 0.087 |
|  | *aprB* | Adenylylsulfate reductase, subunit B | 85 (13) | 87 (3) | 0.605 |
|  | *dsrA* | Dissimilatory sulfite reductase alpha subunit | 598 (27) | 634 (36) | **0.011** |
|  | *dsrB* | Dissimilatory sulfite reductase beta subunit | 288 (35) | 260 (8) | 0.127 |
|  | *dsrC* | Dissimilatory sulfite reductase related protein | 125 (11) | 130 (13) | 0.256 |
|  | *dsrD* | Protein DsrD | 7 (4) | 5 (2) | 0.089 |
|  | *dsrE* | Sulfurtransferase | 25 (7) | 42 (8) | **0.046** |
|  | *dsrF* | Intracellular sulfur oxidation protein | 27 (6) | 38 (3) | **0.018** |
|  | *dsrH* | Intracellular sulfur oxidation protein | 17 (6) | 22 (7) | 0.307 |
|  | *dsrJ* | Protein DsrJ | 21 (5) | 20 (2) | 0.621 |
|  | *dsrK* | Protein DsrK | 485 (22) | 461 (43) | 0.258 |
|  | *dsrL* | Protein DsrL | 128 (9) | 147 (13) | 0.098 |
|  | *dsrM* | Protein DsrM | 219 (8) | 191 (11) | **0.013** |
|  | *dsrN* | Protein DsrN | 313 (24) | 395 (37) | **0.022** |
|  | *dsrO* | Protein DsrO | 45 (4) | 41 (8) | 0.409 |
|  | *dsrP* | Protein DsrP | 67 (9) | 100 (21) | **0.030** |
|  | *dsrT* | Protein DsrT | 36 (7) | 25 (7) | 0.069 |
|  | *qmoA* | Quinone-modifying oxidoreductase, subunit QmoA | 184 (15) | 197 (21) | 0.324 |
|  | *qmoB* | Quinone-modifying oxidoreductase, subunit QmoB | 369 (49) | 352 (31) | 0.396 |
|  | *qmoC* | Quinone-modifying oxidoreductase, subunit QmoC | 104 (16) | 67 (15) | **0.004** |
|  | *rdsr* | Reverse dissimilatory sulfite reductase | 16 (8) | 20 (7) | 0.056 |
|  | *sat* | Sulfate adenylyltransferase | 1284 (39) | 1363 (34) | **0.013** |
| Sulfur reduction | *asrA* | Anaerobic sulfite reductase subunit A | 216 (19) | 257 (11) | **0.037** |
|  | *asrB* | Anaerobic sulfite reductase subunit B | 459 (21) | 393 (60) | **0.026** |
|  | *asrC* | Anaerobic sulfite reductase subunit C | 171 (13) | 173 (6) | 0.841 |
|  | *fsr* | Sulfite reductase (coenzyme F420) | 7 (3) | 7 (2) | 1.000 |
|  | *hydA* | Sulfhydrogenase subunit alpha | 60 (6) | 57 (14) | 0.778 |
|  | *hydB* | Sulfhydrogenase subunit beta (sulfur reductase) | 62 (10) | 50 (8) | 0.107 |
|  | *hydD* | Sulfhydrogenase subunit delta | 268 (19) | 265 (29) | 0.859 |
|  | *hydG* | Sulfhydrogenase subunit gamma (sulfur reductase) | 5 (2) | 3 (1) | 0.121 |
|  | *mccA* | Dissimilatory sulfite reductase | 2 (1) | 1 (1) | 0.145 |
|  | *otr* | Octaheme tetrathionate reductase Otr | 273 (7) | 335 (45) | 0.037 |
|  | *psrA* | Polysulfide reductase chain A | 103 (14) | 91 (8) | 0.184 |
|  | *psrB* | Polysulfide reductase chain B | / | / |  |
|  | *psrC* | Polysulfide reductase chain C | 15 (2) | 13 (3) | 0.416 |
|  | *rdlA* | Putative rhodanese-like protein | 8 (3) | 15 (6) | 0.056 |
|  | *shyA* | Sulfhydrogenase 2 subunit alpha | 301 (22) | 302 (32) | 0.952 |
|  | *shyB* | Sulfhydrogenase 2 subunit beta | 40 (4) | 39 (5) | 0.812 |
|  | *shyC* | Sulfhydrogenase 2 subunit gamma | 375 (30) | 398 (33) | **0.037** |
|  | *shyD* | Sulfhydrogenase 2 subunit delta | 185 (22) | 186 (12) | 0.977 |
|  | *sreA* | Sulfur reductase molybdopterin subunit | 4 (2) | 4 (1) | 0.729 |
|  | *sreB* | Sulfur reductase FeS subunit | / | / |  |
|  | *sreC* | Sulfur reductase membrane anchor | / | / |  |
|  | *sudA* | Sulfide dehydrogenase subunit alpha | 7966 (245) | 7539 (293) | 0.051 |
|  | *sudB* | Sulfide dehydrogenase subunit beta | 678 (32) | 616 (56) | 0.059 |
|  | *ttrA* | Tetrathionate reductase subunit A | 380 (24) | 388 (13) | 0.503 |
|  | *ttrB* | Tetrathionate reductase subunit B | 1717 (44) | 1518 (54) | **0.001** |
|  | *ttrC* | Tetrathionate reductase subunit C | 33 (3) | 43 (9) | 0.091 |
| SOX systems | *soxA* | L-cysteine S-thiosulfotransferase | 159 (26) | 197 (32) | 0.147 |
|  | *soxB* | S-sulfosulfanyl-L-cysteine sulfohydrolase | 625 (51) | 766 (68) | **0.019** |
|  | *soxC* | Sulfane dehydrogenase subunit SoxC | 285 (16) | 347 (33) | **0.007** |
|  | *soxD* | S-disulfanyl-L-cysteine oxidoreductase SoxD | 114 (22) | 140 (25) | 0.067 |
|  | *soxX* | L-cysteine S-thiosulfotransferase | 296 (30) | 333 (33) | 0.066 |
|  | *soxY* | Sulfur-oxidizing protein SoxY | 213 (14) | 249 (18) | **0.003** |
|  | *soxZ* | Sulfur-oxidizing protein SoxZ | 68 (17) | 100 (5) | **0.012** |
| Sulfur oxidation | *doxA* | thiosulfate dehydrogenase [quinone] small subunit | / | / |  |
|  | *doxD* | thiosulfate dehydrogenase [quinone] large subunit | / | / |  |
|  | *fccA* | cytochrome subunit of sulfide dehydrogenase | 94 (14) | 158 (34) | **0.034** |
|  | *fccB* | Sulfide dehydrogenase [flavocytochrome c] flavoprotein chain | 391 (53) | 523 (79) | **0.047** |
|  | *glpE* | Thiosulfate sulfurtransferase | 3786 (155) | 4283 (66) | **0.004** |
|  | *soeA* | Sulfite dehydrogenase (quinone) subunit SoeA | 319 (42) | 435 (29) | **0.021** |
|  | *soeB* | Sulfite dehydrogenase (quinone) subunit SoeB | 11 (3) | 17 (7) | 0.106 |
|  | *soeC* | Sulfite dehydrogenase (quinone) subunit SoeC | 79 (10) | 116 (22) | **0.024** |
|  | *sorA* | Sulfite cytochrome c oxidoreductase-subunitA | 137 (9) | 168 (15) | **0.014** |
|  | *sorB* | Sulfite cytochrome c oxidoreductase-subunitB | 29 (5) | 33 (4) | 0.233 |
|  | *sqr* | Sulfide:quinone oxidoreductase | 176 (20) | 284 (34) | **0.001** |
|  | *sseA* | Thiosulfate sulfurtransferase | 470 (28) | 641 (39) | **0.001** |
|  | *tsdA* | Thiosulfate dehydrogenase | 827 (65) | 854 (25) | 0.296 |
|  | *tsdB* | Thiosulfate dehydrogenase electron acceptor | 182 (32) | 222 (29) | 0.050 |
| Sulfur disproportionation | *phsA* | Thiosulfate reductase | 274 (21) | 287 (33) | 0.338 |
|  | *phsB* | Thiosulfate reductase electron transport protein | 63 (9) | 60 (7) | 0.564 |
|  | *phsC* | Thiosulfate reductase cytochrome b subunit | 127 (8) | 122 (9) | 0.565 |
|  | *tetH* | Tetrathionate hydrolase TetH | 3 (2) | 2 (2) | 0.576 |
|  | *sor* | Sulfur oxygenase/reductase | 4 (1) | 5 (1) | 0.235 |
| Organic sulfur transformation | *acuI* | Acrylyl-CoA reductase AcuI | 1731 (217) | 2053 (158) | **0.030** |
|  | *acuK* | Enoyl-CoA hydratase/isomerases AcuK | 15 (5) | 10 (3) | 0.122 |
|  | *acuN* | CoA-transferase family III AcuN | 43 (7) | 39 (6) | 0.182 |
|  | *betA* | Oxygen-dependent choline dehydrogenase | 1938 (161) | 2332 (108) | **0.001** |
|  | *betB* | NAD/NADP-dependent betaine aldehyde dehydrogenase | 4402 (404) | 5094 (323) | **0.001** |
|  | *betC* | Choline-sulfatase | 4109 (381) | 4037 (432) | 0.815 |
|  | *comA* | Phosphosulfolactate synthase | 47 (5) | 59 (12) | 0.188 |
|  | *comB* | 2-phosphosulfolactate phosphatase | 420 (20) | 469 (26) | **0.002** |
|  | *comC* | L-sulfolactate dehydrogenase | 154 (19) | 131 (14) | **0.006** |
|  | *comD* | Sulfopyruvate decarboxylase subunit alpha | 47 (10) | 34 (5) | 0.075 |
|  | *comE* | Sulfopyruvate decarboxylase subunit beta | 82 (11) | 93 (14) | 0.178 |
|  | *dddA* | Glucose-methanol-choline flavoprotein oxido-reductases DddA | 2 (2) | 1 (1) | 0.338 |
|  | *dddC* | NADP-containing dehydrogenases DddC | 9 (2) | 7 (4) | 0.255 |
|  | *dddD* | CoA-transferase/lyase DddD | 31 (7) | 26 (7) | 0.209 |
|  | *dddK* | Dimethlysulfonioproprionate lyase | / | / |  |
|  | *dddL* | Dimethlysulfonioproprionate lyase | / | / |  |
|  | *dddP* | Dimethlysulfonioproprionate lyase DddP | 218 (15) | 268 (15) | **0.008** |
|  | *dddQ* | Dimethlysulfonioproprionate lyase DddQ | / | / |  |
|  | *dddT* | Betaine/carnitine/choline transporter DddT | 11 (6) | 30 (7) | **0.008** |
|  | *dddW* | Dimethlysulfonioproprionate lyase DddW | 5 (1) | 5 (4) | 1.000 |
|  | *dddY* | Dimethlysulfonioproprionate lyase DddY | / | / |  |
|  | *ddhA* | Dimethylsulfide dehydrogenase subunit alpha | 132 (22) | 68 (17) | **0.001** |
|  | *ddhB* | Dimethylsulfide dehydrogenase subunit beta | 3 (1) | 2 (2) | 0.099 |
|  | *ddhC* | Dimethylsulfide dehydrogenase subunit gamma | / | / |  |
|  | *dmdA* | Dimethylsulfoniopropionate demethylase | 109 (12) | 114 (12) | 0.130 |
|  | *dmdB* | 3-(methylthio)propionyl---CoA ligase | 3007 (202) | 3080 (83) | 0.304 |
|  | *dmdC* | 3-(methylthio)propanoyl-CoA dehydrogenase | 4005 (173) | 4035 (109) | 0.685 |
|  | *dmdD* | (methylthio)acryloyl-CoA hydratase | 1583 (148) | 1569 (90) | 0.805 |
|  | *dmoA* | dimethyl-sulfide monooxygenase | 15 (5) | 27 (2) | **0.003** |
|  | *dmsA* | Anaerobic dimethyl sulfoxide reductase subunit A | 3674 (89) | 3542 (140) | 0.079 |
|  | *dmsB* | Anaerobic dimethyl sulfoxide reductase subunit B | 1557 (80) | 1372 (78) | 0.050 |
|  | *dmsC* | Anaerobic dimethyl sulfoxide reductase subunit C | 1065 (48) | 1080 (23) | 0.599 |
|  | *dsyB* | DsyB | 263 (27) | 308 (23) | **0.021** |
|  | *gdh* | Glutamate dehydrogenase (NADP+) | 1284 (35) | 1211 (67) | 0.167 |
|  | *hpsN* | Sulfopropanediol 3-dehydrogenase | 750 (26) | 844 (16) | **0.001** |
|  | *hpsO* | R or S-dihydroxypropanesulfonate-2-dehydrogenase | 138 (18) | 150 (16) | **0.033** |
|  | *hpsP* | R or S-dihydroxypropanesulfonate-2-dehydrogenase | 724 (25) | 774 (44) | 0.134 |
|  | *iseJ* | Isethionate dehydrogenase | / | / |  |
|  | *isfD* | Sulfoacetaldehyde reductase | 2045 (97) | 2323 (130) | **0.001** |
|  | *mddA* | Methanethiol S-methyltransferase | 88 (9) | 116 (18) | 0.043 |
|  | *mdh* | Malate dehydrogenase | 4177 (69) | 4423 (128) | **0.008** |
|  | *mtsA* | Methylthiol:coenzyme M methyltransferase | 35 (7) | 25 (6) | **0.034** |
|  | *mtsB* | Methylated-thiol--corrinoid protein | 575 (29) | 583 (53) | 0.753 |
|  | *prpE* | Propionate--CoA ligase | 3674 (125) | 3903 (165) | 0.114 |
|  | *pta* | Phosphate acetyltransferase | 4113 (238) | 4403 (160) | 0.156 |
|  | *sfnG* | Dimethylsulfone monooxygenase | 26 (9) | 34 (11) | 0.315 |
|  | *slcC* | s-sulfolactate dehydrogenase | 2074 (40) | 2154 (52) | **0.049** |
|  | *slcD* | Sulfolactate dehydrogenase SlcD | 136 (21) | 184 (27) | **0.028** |
|  | *sqdB* | UDP-sulfoquinovose synthase | 654 (21) | 670 (19) | 0.280 |
|  | *sqdD* | Glycosyltransferase | / | / |  |
|  | *sqdX* | Glycogen synthase | 1690 (58) | 2008 (68) | **0.001** |
|  | *tauX* | Taurine dehydrogenase small subunit | 13 (3) | 19 (3) | 0.060 |
|  | *tauY* | Taurine dehydrogenase large subunit | 16 (6) | 27 (8) | **0.004** |
|  | *tmm* | Trimethylamine monooxygenase | 21 (6) | 35 (5) | **0.040** |
|  | *toa* | Taurine:2-oxoglutarate transaminase | 301 (31) | 328 (33) | 0.138 |
|  | *tpa* | Taurine-pyruvate aminotransferase | 2606 (151) | 2702 (105) | 0.170 |
|  | *yihQ* | Sulfoquinovosidase | 114 (13) | 141 (10) | **0.037** |
| Link between inorganic and organic sulfur transformation | *cuyA* | L-cysteate sulfo-lyase | 462 (29) | 459 (23) | 0.843 |
|  | *cysE* | Serine O-acetyltransferase | 2486 (95) | 2635 (78) | **0.018** |
|  | *cysK* | Cysteine synthase | 4566 (136) | 4962 (170) | **0.002** |
|  | *cysM* | Cysteine synthase | 1569 (63) | 1706 (50) | **0.004** |
|  | *cysO* | Cysteine synthase | / | / |  |
|  | *hdrA1* | heterodisulfide reductase subunit A1 | 1046 (120) | 864 (72) | **0.007** |
|  | *hdrA2* | heterodisulfide reductase subunit A2 | 3130 (393) | 2371 (333) | **0.011** |
|  | *hdrB1* | heterodisulfide reductase subunit B1 | 144 (25) | 141 (17) | 0.809 |
|  | *hdrB2* | heterodisulfide reductase subunit B2 | 248 (37) | 219 (30) | 0.105 |
|  | *hdrC1* | heterodisulfide reductase subunit C1 | 35 (6) | 36 (4) | 0.737 |
|  | *hdrC2* | heterodisulfide reductase subunit C2 | 231 (32) | 207 (40) | 0.315 |
|  | *hdrD* | heterodisulfide reductase subunit D | 1536 (131) | 1389 (141) | **0.014** |
|  | *hdrE* | heterodisulfide reductase subunit E | 3 (2) | 3 (1) | 0.541 |
|  | *mccB* | Cystathionine gamma-lyase / homocysteine desulfhydrase | 284 (33) | 316 (36) | 0.064 |
|  | *metA* | Homoserine O-succinyltransferase/ O-acetyltransferase | 168 (10) | 229 (11) | **0.003** |
|  | *metB* | Cystathionine gamma-synthase | 1280 (99) | 1394 (67) | 0.065 |
|  | *metC* | Cystathionine beta-lyase | 1979 (138) | 2136 (80) | **0.031** |
|  | *metX* | Homoserine O-acetyltransferase/O-succinyltransferase | 831 (54) | 892 (100) | 0.249 |
|  | *metY* | O-acetyl-L-homoserine sulfhydrylase | 1043 (70) | 1141 (63) | 0.067 |
|  | *metZ* | O-succinylhomoserine sulfhydrylase | 1004 (65) | 1166 (54) | **0.004** |
|  | *msmA* | Methanesulfonate monooxygenase subunit alpha | 74 (7) | 128 (29) | **0.010** |
|  | *msmB* | Methanesulfonate monooxygenase subunit beta | / | / |  |
|  | *mtoX* | Methanethiol oxidase | 8 (3) | 11 (4) | 0.377 |
|  | *ssuD* | Alkanesulfonate monooxygenase | 1615 (119) | 1860 (61) | **0.003** |
|  | *ssuE* | FMN reductase | 201 (23) | 252 (35) | 0.063 |
|  | *suyA* | (2R)-sulfolactate sulfo-lyase subunit alpha | 266 (30) | 258 (44) | 0.764 |
|  | *suyB* | (2R)-sulfolactate sulfo-lyase subunit beta | 208 (16) | 202 (23) | 0.560 |
|  | *tauD* | Taurine dioxygenase | 864 (41) | 997 (49) | **0.001** |
|  | *tbuB* | Toluene-3-monooxygenase | 7 (3) | 11 (5) | 0.258 |
|  | *tbuC* | Toluene-3-monooxygenase | 76 (19) | 98 (14) | 0.091 |
|  | *tmoC* | Toluene-4-monooxygenase | 46 (9) | 80 (9) | **0.002** |
|  | *tmoF* | Toluene-4-monooxygenase | 4 (1) | 5 (4) | 0.546 |
|  | *touC* | Toluene o-xylene monooxygenase | / | / |  |
|  | *touF* | Toluene o-xylene monooxygenase | 17 (3) | 23 (1) | **0.005** |
|  | *xsc* | Sulfoacetaldehyde acetyltransferase | 1582 (61) | 1626 (54) | 0.238 |
| Others | *cuyZ* | Sulfite exporter | / | / |  |
|  | *cysA* | Sulfate/thiosulfate import ATP-binding protein CysA | 2801 (107) | 2962 (125) | **0.045** |
|  | *cysP* | Thiosulfate-binding protein | 510 (27) | 505 (24) | 0.766 |
|  | *cysU* | Sulfate transport system permease protein CysT | 1414 (94) | 1692 (11) | **0.002** |
|  | *cysW* | Sulfate transport system permease protein CysW | 371 (32) | 439 (17) | **0.004** |
|  | *cysZ* | Sulfate transporter CysZ | 85 (8) | 99 (14) | 0.080 |
|  | *hpsK* | Dihydroxypropanesulfonate transporter | 34 (5) | 35 (3) | 0.582 |
|  | *hpsL* | Dihydroxypropanesulfonate transporter | 13 (5) | 9 (2) | 0.167 |
|  | *hpsM* | Dihydroxypropanesulfonate transporter | 193 (9) | 184 (17) | 0.367 |
|  | *iseK* | Isethionate TRAP transporter | / | / |  |
|  | *iseL* | Isethionate TRAP transporter | / | / |  |
|  | *iseM* | Isethionate TRAP transporter | 1051 (31) | 961 (47) | **0.015** |
|  | *sbp* | Sulfate-binding protein | 361 (21) | 402 (28) | **0.022** |
|  | *sgpA* | Sulfur globule protein CV1 | 3 (1) | 5 (2) | 0.167 |
|  | *sgpB* | Sulfur globule protein CV2 | 135 (17) | 136 (9) | 0.929 |
|  | *sgpC* | Sulfur globule protein CV3 | 39 (10) | 54 (8) | **0.005** |
|  | *soxL* | Sulfur transferase, periplasm | 4 (1) | 6 (2) | 0.161 |
|  | *ssuA* | Sulfonate transport system substrate-binding protein | 5276 (364) | 4761 (85) | 0.052 |
|  | *ssuB* | Sulfonate transport system ATP-binding protein | 2729 (121) | 2885 (65) | **0.019** |
|  | *ssuC* | Sulfonate transport system permease protein | 1756 (85) | 1968 (90) | **0.003** |
|  | *sulP* | Sulfate permease | 3002 (171) | 3681 (224) | **0.010** |
|  | *tauA* | Taurine transport system substrate-binding protein | 346 (28) | 372 (47) | 0.472 |
|  | *tauB* | Taurine transport system ATP-binding protein | 1120 (63) | 1264 (39) | **0.001** |
|  | *tauC* | Taurine transport system permease protein | 135 (9) | 133 (22) | 0.847 |
|  | *tauE* | Sulfite/organosulfonate exporter | 7 (5) | 9 (3) | 0.467 |
|  | *tauZ* | Membrane protein TauZ | 47 (6) | 63 (9) | **0.001** |
|  | *tusA* | Sulfur carrier protein TusA | 626 (38) | 675 (25) | 0.081 |
|  | *tusB* | tRNA 2-thiouridine synthesizing protein B | 285 (22) | 379 (6) | **0.001** |
|  | *tusC* | tRNA 2-thiouridine synthesizing protein C | 10 (4) | 15 (3) | 0.154 |
|  | *tusD* | Sulfurtransferase TusD | 173 (25) | 206 (9) | 0.054 |
|  | *tusE* | Sulfurtransferase TusE | 227 (9) | 311 (18) | **0.001** |

Data are presented as mean (Standard error); /: either not detected in our data, or the average number of sequences was less than 1. *p* value was obtained by two-sample Student's t test.
