# Supplementary material for: Metagenomic insights into the effects of submerged plants on functional potential of microbial communities in wetland sediments
Source: Mar Life Sci Technol. 2021 Aug 27;3(4):405–15. doi: 10.1007/s42995-021-00100-3 (PMC10077182; doi:10.1007/s42995-021-00100-3)
Supplement: Supplementary file 4 — Supplementary file4 (DOCX 24 KB) [file 42995_2021_100_MOESM4_ESM.docx]

Summary of representative sequences for selected gene families in methanogenesis

| Pathway | Gene（sub）family | Annotation | Representative sequences | | |
| --- | --- | --- | --- | --- | --- |
|  |  |  | No plants | Submerged plants | *p* |
| Core carbon | *meh* | 3-methylfumaryl-CoA hydratase | 77 (17) | 83 (21) | 0.680 |
|  | *prkB* | phosphoribulokinase | 169 (14) | 232 (40) | **0.012** |
|  | *rbcL* | ribulose-bisphosphate carboxylase large chain | 433 (34) | 554 (77) | **0.012** |
|  | *coxS* | aerobic carbon-monoxide dehydrogenase small subunit | 681 (46) | 691 (60) | 0.783 |
|  | *coxM* | aerobic carbon-monoxide dehydrogenase medium subunit | 554 (21) | 596 (62) | 0.185 |
|  | *coxL* | aerobic carbon-monoxide dehydrogenase large subunit | 718 (64) | 1004 (236) | **0.031** |
|  | *abfD* | 4-hydroxybutyryl-CoA dehydratase / vinylacetyl-CoA-Delta-isomerase | 546 (83) | 439 (84) | 0.076 |
|  | *acs* | acetyl-CoA synthetase | 2955 (76) | 3177 (131) | **0.011** |
|  | *acsB* | acetyl-CoA synthase | 630 (27) | 511 (42) | **0.001** |
|  | *coxA* | cytochrome c oxidase subunit I | 1214 (96) | 1388 (120) | **0.036** |
|  | *coxC* | cytochrome c oxidase subunit III | 444 (44) | 527 (62) | **0.041** |
|  | *korA* | 2-oxoglutarate/2-oxoacid ferredoxin oxidoreductase subunit alpha | 2737 (93) | 2532 (126) | **0.019** |
|  | *korB* | 2-oxoglutarate/2-oxoacid ferredoxin oxidoreductase subunit beta | 1693 (18) | 1555 (64) | **0.002** |
|  | *frdA* | fumarate reductase flavoprotein subunit | 656 (38) | 683 (68) | 0.460 |
|  | *ldh* | L-lactate dehydrogenase | 169 (13) | 215 (20) | **0.003** |
|  | *K00400/mcrA2* | methyl coenzyme M reductase system, component A2 | 63 (20) | 106 (23) | **0.014** |
| Carbon dioxide | *mtrA* | tetrahydromethanopterin S-methyltransferase subunit A | 95 (19) | 117 (31) | 0.215 |
|  | *fmdA* | formylmethanofuran dehydrogenase subunit A | 165 (50) | 223 (36) | 0.069 |
|  | *fmdB* | formylmethanofuran dehydrogenase subunit B | 148 (51) | 191 (31) | 0.146 |
|  | *mer* | 5,10-methylenetetrahydromethanopterin reductase | 156 (14) | 212 (32) | **0.007** |
|  | *ftr* | formylmethanofuran--tetrahydromethanopterin N-formyltransferase | 102 (40) | 122 (33) | 0.400 |
|  | *fmdE* | formylmethanofuran dehydrogenase subunit E | 261 (33) | 244 (34) | 0.466 |
| Core methanogenesis | *mcrA* | methyl-coenzyme M reductase alpha subunit | 51 (13) | 81 (29) | 0.077 |
|  | *hdrA2* | heterodisulfide reductase subunit A2 | 4219 (550) | 3267 (403) | **0.014** |
|  | *hdrB2* | heterodisulfide reductase subunit B2 | 804 (79) | 775 (71) | 0.564 |
|  | *hdrC2* | heterodisulfide reductase subunit C2 | 414 (52) | 399 (34) | 0.585 |
|  | *hdrD* | heterodisulfide reductase subunit D | 579 (88) | 491 (64) | 0.107 |
|  | *mvhA* | F420-non-reducing hydrogenase large subunit | 723 (56) | 606 (56) | **0.011** |
|  | *mvhG* | F420-non-reducing hydrogenase small subunit | 493 (29) | 395 (30) | **0.001** |
| Acetate | *cdhA* | anaerobic carbon-monoxide dehydrogenase, CODH/ACS complex subunit alpha | 105 (44) | 85 (23) | 0.381 |
|  | *cdhC* | acetyl-CoA decarbonylase/synthase, CODH/ACS complex subunit beta | 118 (39) | 116 (22) | 0.891 |
|  | *cdhE* | acetyl-CoA decarbonylase/synthase, CODH/ACS complex subunit gamma | 628 (58) | 568 (63) | 0.152 |
| Methylamine | *mttB* | trimethylamine---corrinoid protein Co-methyltransferase | 1293 (95) | 1524 (73) | **0.003** |
|  | *mtmB* | methylamine---corrinoid protein Co-methyltransferase | 72 (21) | 95 (13) | 0.075 |
| Methanol | *mtaA* | [methyl-Co(III) methanol-specific corrinoid protein]:coenzyme M methyltransferase | 136 (17) | 109 (15) | **0.029** |

Data are presented as mean (Standard error); *p* value was obtained by two-sample Student's t test.
